# NOX2/NOX4-associated oxidative stress contributes to G6PD-associated metabolic remodeling and ferroptosis during mice luteolysis

**DOI:** 10.1080/13510002.2026.2724653

**Published:** 2026-09-04

**Authors:** Fei Li, JiaHao Song, Wei Zou, Yulu Zhang, YanMin Cheng, HongRu Zhao, Bei Yang, Haibin Kuang

**Affiliations:** a Department of Physiology, School of Basic Medical Sciences; School of Nursing, Jiangxi Medical College, Nanchang University, Jiangxi, People's Republic of China; b Jiangxi Provincial Key Laboratory of Disease Prevention and Public Health, Nanchang University, Jiangxi, People's Republic of China

**Keywords:** Luteolysis, metabolic remodeling, NOX2/NOX4, G6PD, ferroptosis, redox imbalance, oxidative stress, pentose phosphate pathway

## Abstract

**Objective:**

Ferroptosis has been shown to participate in luteolysis, yet the upstream signals and metabolic remodeling that trigger it remain unknown. This study aims to clarify these mechanisms.

**Methods:**

Physiological postpartum and PGF2α-induced luteolysis models were established in mice. Targeted energy metabolomics, pharmacological inhibition and adenoviral shRNA-mediated knockdown were employed in vivo and in primary luteal cells to measure ferroptosis markers, G6PD activity, NADP⁺/NADPH ratio and NOX2/NOX4 expression.

**Results:**

In both models, luteal regression was accompanied by increased ACSL4, decreased GPx4 and GSH, and elevated Fe^2^⁺ and lipid peroxidation and NOX2 and NOX4 expression. Metabolic analysis revealed 6-phosphogluconate accumulation, reduced pyruvate and pyruvate kinase M activity, and increased G6PD expression and activity, but NADPH depletion and redox imbalance. *In vivo*, combined NOX2/NOX4 knockdown reduced ROS accumulation, suppressed G6PD hyperactivation, alleviated ferroptotic injuryand delayed regression Moreover, G6PD inhibition with 6-AN similarly reduced oxidative stress and ferroptosis and partially restored progesterone secretion. In primary luteal cells, G6PD knockdown or 6-AN treatment attenuated PGF2α-induced ferroptosis and improved cell viability. Simultaneously, NOX2/NOX4 silencing, N-acetylcysteine, or Ferrostatin-1 suppressed PGF2α-induced G6PD activation, and attenuated oxidative stress and ferroptosis.

**Conclusion:**

NOX2/NOX4-associated oxidative stress contributes to G6PD-associated metabolic remodeling and ferroptosis during mice luteolysis.

## Graphical abstract

**Figure:**
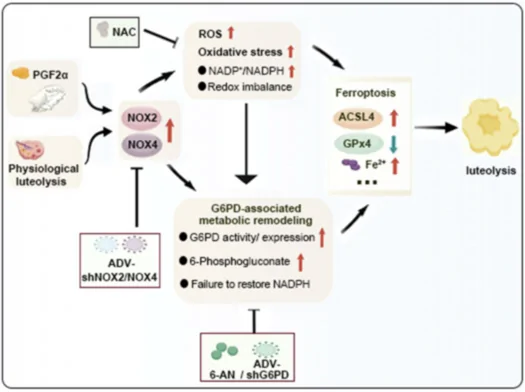

This graphical abstract summarizes the central mechanism proposed in this study.

During PGF2α-induced and physiological luteolysis, NOX2/NOX4 expression is increased, promoting ROS accumulation and disruption of the NADP⁺/NADPH redox balance, thereby aggravating ferroptosis-related injury. Sustained oxidative stress further induces G6PD-associated metabolic remodeling, characterized by increased G6PD expression and activity and the accumulation of 6-phosphogluconate. However, these changes fail to restore NADPH homeostasis, and NADPH levels remain reduced. Combined knockdown of NOX2/NOX4 or inhibition of G6PD alleviates these oxidative–metabolic disturbances and ferroptotic damage. These findings indicate that G6PD hyperactivation represents a maladaptive metabolic response to sustained oxidative stress and functionally contributes to ferroptosis-related injury and the progression of luteolysis.

## 1. Introduction

The corpus luteum is a transient endocrine gland formed after ovulation that sustains early pregnancy primarily through progesterone secretion [1–4]. Its function depends on the tightly coordinated processes of formation, maintenance, and regression. Luteolysis involves both functional regression, manifested by reduced progesterone secretion, and structural regression, characterised by luteal cell loss [1,5,6]. Accordingly, increasing attention has focused on the molecular mechanisms governing luteolysis, particularly the regulated cell-death pathways involved in structural luteal regression [7–9].

Regulated cell death is a central event in corpus luteum regression, which involves multiple death-related mechanisms, including apoptosis, autophagy, and necroptosis [6,10–12]. This complexity has prompted increasing attention to other regulated cell-death mechanisms. Ferroptosis is an iron-dependent form of regulated cell death driven by the uncontrolled peroxidation of membrane phospholipids and is associated with iron accumulation, impaired glutathione-dependent antioxidant defence, redox imbalance [13–15]. These features are closely related to the intense steroidogenic activity, active lipid metabolism, and rapid structural remodelling of the corpus luteum. Our previous study identified ferroptosis as a key mechanism involved in luteal regression [16]. However, the molecular events that regulate ferroptosis during luteolysis remain unclear.

Ferroptosis is not only a form of regulated cell death but also a metabolically conditioned process, as its sensitivity is shaped by lipid metabolism, iron handling, antioxidant defence, and redox homoeostasis [13,14]. Energy metabolism, including glycolysis, the pentose phosphate pathway (PPP), and the tricarboxylic acid cycle, can substantially influence ferroptosis sensitivity. For instance, metabolic stress or inhibition of glycolysis can suppress ferroptosis through AMPK activation [17]. Among these metabolic pathways, the oxidative PPP is particularly relevant because it generates NADPH, which supports glutathione-dependent antioxidant defence and influences cellular ROS levels and lipid peroxidation [18–22]. However, whether luteolysis is accompanied by coordinated remodelling of energy metabolism, and which metabolic pathways or regulatory nodes contribute to ferroptosis during this process, remain unclear.

Among the redox-regulatory pathways potentially relevant to luteolysis, NADPH oxidases (NOXs) are of particular interest because they use NADPH as an electron donor to generate ROS [23]. Evidence from ovarian cells indicates that NOX4 is expressed in granulosa cells and can function as an important source of H₂O₂ in human granulosa-lutein cells [24,25]. Moreover, PGF2α has been reported to induce NADPH oxidase signalling through NOX1 in vascular smooth muscle cells, suggesting that PGF2α may engage NOX-dependent redox signalling in a non-ovarian cellular context [26]. NOX-derived ROS have been implicated in lipid peroxidation, iron dysregulation, and ferroptosis [23,27–30], and can also influence glucose and lipid metabolism [31,32]. However, whether specific NOX isoforms contribute to oxidative stress, ferroptosis, and metabolic remodelling during luteolysis remains unknown.

A notable observation from our initial analyses was the marked increase in glucose-6-phosphate dehydrogenase (G6PD) expression and activity, but markedly reduced NADPH levels. This pattern was consistent with an imbalance between NADPH production and consumption, rather than effective restoration of redox homoeostasis. Although studies as early as 1976 reported increased G6PD activity during luteolysis [33], the upstream signals driving this response and its functional consequences during luteal regression have remained poorly understood.

Accordingly, the present study aimed to characterise alterations in energy metabolism during luteolysis and to investigate the interplay among NOX-associated oxidative stress, G6PD activation, and ferroptosis. We combined a physiological postpartum luteolysis model with a temporally controllable PGF2α-induced luteolysis model and integrated targeted energy metabolomics with *in vivo* and *in vitro* loss-of-function approaches. Our findings indicate that NOX2/NOX4-associated oxidative stress contributes to G6PD hyperactivation and ferroptotic injury during luteolysis, while G6PD inhibition attenuates these changes and partially preserves luteal steroidogenic function *in vivo*.

## 2. Materials and methods

### 2.1. Reagents

Fer-1 (Cat. HY-100579, MCE), PGF2α (Sigma-Aldrich, Cat. P5069), human chorionic gonadotrophin (HCG, GLPBIO, Cat. GP21266), pregnant mare serum gonadotropin (PMSG, GLPBIO, Cat. GP21284), *N*-acetylcysteine (NAC, MCE, Cat. HY-B0215), 6-AN (MCE, Cat. HY-W010342) were used in the experiments.

### 2.2. Animal treatment

All animal experiments were approved by the Animal Ethics Committee of Nanchang University (NO: NCULAE-202206240141). All mice were subjected to experiments under deep anaesthesia induced by intraperitoneal injection of pentobarbital (40mg/kg), and those undergoing retro-orbital blood collection or ovarian tissue harvest were euthanized by cervical dislocation while remaining fully anaesthetised immediately after the procedures.

Sexually mature female Kunming mice (6 weeks old, 30 ± 2 g) and immature mice (21 days old, 15 ± 2 g) were obtained from Jiangxi University of Traditional Chinese Medicine (Jiangxi, China). Mice were housed under standard conditions with a 12h light/dark cycle and controlled environmental temperature and humidity. Food and water were provided ad libitum. Mature females were paired with fertile males at a 2:1 ratio, and pregnancy was confirmed by detecting a vaginal plug. As reported previously, luteal development peaks around gestation day 9 (GD9), followed by pronounced regression during late pregnancy and the early postpartum period [34,35]. Ovarian tissues were collected at GD9 and postpartum day 1 (PP1). The GD9-PP1 comparison was used to reflect physiological postpartum luteal regression in mice.

For the construction of the PGF2α-induced luteolysis model, 5 IU PMSG was administered intraperitoneally to immature mice (25-day-old), followed 48h later by 5 IU hCG [36] to induce ovulation. At 72h after hCG, 5mg/kg PGF2α (dissolved in saline) was injected intraperitoneally to induce luteolysis [37,38], and the control group received an equal volume of saline. Additionally, to establish G6PD inhibition model, PGF2α was co-administered with 30mg/kg 6-AN [39] (a selective G6PD inhibitor dissolved in phosphate-buffered saline (PBS)) at the same time point. This minimal effective dose was determined by in vivo dose-gradient screening, achieving robust ovarian G6PD inhibition with minimal systemic toxicity, consistent with previous reports. Inhibition efficacy was validated by enzymatic activity assay (Supplementary Figure 6C). The PGF2α+Vehicle group was injected with PGF2α (in saline) plus an equal volume of PBS (used as the vehicle for 6-AN). Ovaries were harvested 24h after PGF2α injection.

### 2.3. HE staining

Paraffin sections of ovarian tissue were deparaffinized in xylene solution, rehydrated with a graded ethanol series, and stained with hematoxylin for 5min and eosin for 2min. Sections were then dehydrated, cleared in xylene, and mounted.

### 2.4. Immunohistochemistry

After deparaffinization and rehydration, antigen retrieval was performed in citrate buffer (pH 6.0) by microwave heating. Sections were blocked with 3% (v/v) H2O2 and 5% (w/v) BSA and then incubated overnight at 4 °C with primary antibodies against G6PD (Cat. 66373-1-Ig, Proteintech), NOX2 (Cat. 19013-1-AP, Proteintech) and NOX4 (Cat. 14347-1-AP, Proteintech) (1:500 (v/v)). After washing, the sections were incubated with HRP-conjugated goat anti-rabbit IgG (H+L) secondary antibody (Proteintech, Cat. SA00001-2; 1:200) or an HRP-conjugated goat anti-mouse IgG (H+L) secondary antibody (Proteintech, Cat. SA00001-1; 1:200), at 37° C for 1h, as appropriate. Signals were visualised using DAB (Zhongshan Golden Bridge, Cat. ZLI-9018) and counterstained with hematoxylin.

### 2.5. Primary corpus luteum isolation and cell culture

Immature mice at postnatal day 21 (P21) received 5 IU PMSG and 5 IU HCG with a 48-h interval between the injections. On day 4 after ovulation, ovaries were digested with a solution of 1mg/mL collagenase (Beijing Solarbio, Cat. C8140), 60μg/mL DNase I (Beijing Solarbio, Cat. D8075), and 0.25% (w/v) trypsin (Procell, Cat. PB180225) at 37 °C for 3 min repeated three times. The cells were then filtered, centrifuged, and resuspended in DMEM (Solarbio, Cat. 11995) supplemented with 10% (v/v) FBS (Gibco, Cat. A3382001). Luteal cells were stimulated with 1µM PGF2α [16] to induce luteolysis for 12 or 24 hours. The concentration of 1μM PGF2α was selected based on preliminary dose–response experiments identifying the lowest effective concentration that induces measurable cellular responses without excessive cytotoxicity.

For adenoviral infection, cells were incubated with adenoviruses (AD-shNC, AD-shNOX2, AD-shNOX4). For dual-gene knockdown, Ad-shNOX2 and Ad-shNOX4 were co-infected at a multiplicity of infection (MOI) of 60. The target sequences were as follows:

Ad-shNOX2: 5’- TCCATGGAGCTGAACGAATTG -3' (NM_007807.5).

Ad-shNOX4: 5’-AATGGAGAATAATATACTGGC-3' (NM_015760.5).

Scrambled control: 5’-TTCTCCGAACGTGTCACGT-3'.

For adenoviral infection, cells were infected with AD-shNC or AD-shG6PD adenoviruses constructed by Shandong Vigene Biosciences Co., Ltd. (Jinan, China) at a multiplicity of infection (MOI) of 60. The target sequences were as follows:

Ad-shG6PD: 5’-GCTGGATCTAACTTATGGCAA-3' (NM_008062.3).

Scrambled control: 5’-TTCTCCGAACGTGTCACGT-3'.

Cells were treated with graded concentrations of 6-AN (0, 1, 5, 15, and 25μM) for 24h. The optimal concentration (15μM) was selected based on dose–response experiments and used for subsequent mechanistic studies.

### 2.6. Western blot analysis

Cell and tissue proteins were extracted using RIPA (Boster, Cat. AR0102) buffer containing protease and phosphatase inhibitors. Equal amounts of protein were denatured at 95°C for 5min, separated by SDS-PAGE using 10–12.5% (w/v) polyacrylamide gels, and transferred to PVDF membranes (Millipore, Cat. IPVH00010). After blocking with 5% (w/v) non-fat milk (or 5% (w/v) BSA for phospho-proteins), membranes were incubated overnight at 4°C with primary antibodies. After washing, the membranes were incubated at room temperature for 1.5h with an HRP-conjugated goat anti-rabbit IgG (H+L) secondary antibody (Proteintech, Cat. SA00001-2; 1:10,000, v/v) or an HRP-conjugated goat anti-mouse IgG (H+L) secondary antibody (Proteintech, Cat. SA00001-1; 1:10,000, v/v), as appropriate. Signals were visualised using an ECL kit (YiSheng, China, Cat. 36208ES60). Primary antibodies: ACSL4 (Proteintech, Cat. 22401-1-AP), G6PD (Abcam, Cat. ab210702), GPx4 (Abmart, Cat. T56959), NOX2 (Abcam, Cat. ab310337), NOX4 (Proteintech, Cat. 14347-1-AP), 20α-hydroxysteroid dehydrogenase (20αHSD) (Proteintech, Cat. 10978-1-AP), *β*-actin (Proteintech, Cat. 66009-1-Ig), Hexokinase 2 (HK2) (Proteintech, Cat. 22029-1-AP), Pyruvate Kinase Muscle Isozyme 2 (PKM2, Proteintech, Cat. 15822-1-AP): all 1:1000 (v/v).

### 2.7. Real-time quantitative PCR

Total RNA was isolated with the TRIzol solution (Ambion, Cat. 15596-026/018), while cDNA transcription was carried out by PrimeScript RT Kit (TaKaRa, Cat. RR037A). Real-time quantitative PCR was conducted with the SYBR Green Premix Ex Taq II solution (TaKaRa, Cat. RR420) on a Bio-Rad CFX 96 apparatus. Relative expression was normalised by using the 2-ΔΔCt method with 18S housekeeping genes. The sequence and accession code were listed as follows. Nox1: Forward, 5’-CTGCTGTCCTTCTTGAGGGG-3’; Reverse, 5’-TGTTGCTATCCCAGCCAGTG-3’ (NM_172203.2); Nox2: Forward, 5’-ATTCTGGTGTGGTTGGGGCT-3’; Reverse, 5’-ACTTGGATACCTTGGGGCAC-3’ (FJ168469.1); Nox3: Forward, 5’-TCTGAACGAGAGTGGGTCCT-3’; Reverse, 5’-AAGGTGCGGACTGGATTGAG-3’ (NM_198958.2); Nox4: Forward, 5’-TCTCAGGTGTGCATGTAGCC-3’; Reverse, 5’-CGGTAAAGTCTCTCCGCACA-3’ (NM_015760.5); Duox1: Forward, 5’-GGGTTCCACTTAGCTCTGGC-3’; Reverse, 5’-GGTAGGGTTCTTTCAGGGGC-3’ (NM_001099297.1); Duox2: Forward, 5’-CTGGGCTTGTTGTGGTTTCG-3’; Reverse, 5’-AGCCTGGCTATAACTGGGGA-3’ (NM_001362755.1); Rn18s: Forward, 5’-AATCAGGGTTCGATTCCGGA-3’; Reverse, 5’-CCAAGATCCAACTACGAGCT-3’ (NR_003278.3).

### 2.8. In situ ovarian injection of adenoviral vectors

Mice were anaesthetised with pentobarbital sodium and placed under sterile conditions. A dorsal incision was made to expose the ovaries, and adenoviral vectors carrying shRNAs that specifically and separately targeted NOX2 or NOX4 (1× 10^8^ PFU/mL, Shanghai He Yuan Biotechnology Co., Ltd.) were injected directly into the ovary using a microsyringe. Muscles were closed with absorbable sutures and the skin incision was closed with a wound clip. Mice recovered under standard conditions until tissue collection. Target sequences were:

Ad-shNOX2: 5’-TCCATGGAGCTGAACGAATTG-3' (NM_007807.5).

Ad-shNOX4: 5’-AATGGAGAATAATATACTGGC-3' (NM_015760.5).

Scrambled control: 5’-TTCTCCGAACGTGTCACGT-3'.

### 2.9. Measurement of ROS

Ovarian tissues were homogenised in extraction buffer and centrifuged. An aliquot of the supernatant was used to calculate protein concentration. The other supernatant was mixed with assay reagents and transferred to 96-well plates. For cellular ROS assessment, 10,000 cells/well were seeded on 96-well plates, treated as indicated. After washing with PBS, the cells were incubated with DCFH-DA diluted 1:1,000 (v/v) in serum-free medium for 30 min in the dark. Parallel wells were used to assess cell viability by CCK-8 assay. For both tissue and cell samples, fluorescence was measured at excitation and emission wavelengths of 488 and 525 nm, respectively. Tissue ROS analysis kit (Cat. G0163W) was obtained from Suzhou Grace Biotechnology Co., Ltd. (Suzhou, China), and cell ROS analysis kit (Cat. E004-1-1) was obtained from Nanjing Jiancheng Bioengineering Institute (Nanjing, China). All procedures were performed according to the manufacturers' instructions.

### 2.10. Measurement of Fe^2+^, GSH, MDA levels

Tissue or cell samples were homogenised and centrifuged. An aliquot of the supernatant was used to calculate protein concentration. The other supernatant was mixed with assay reagents and transferred to 96-well plates. The absorbance readings were as follows: Fe^2^⁺ at 562 nm, GSH at 412 nm, and MDA at 532 nm. Fe^2^⁺ (Cat. G1217W) and GSH (Cat. G0206W) analysis kits were obtained from Suzhou Grace Biotechnology Co., Ltd. (Suzhou, China), whereas the MDA analysis kit (Cat. A003-2-1) was obtained from Nanjing Jiancheng Bioengineering Institute (Nanjing, China).

### 2.11. Measurement of NADPH and NADP⁺ levels

After homogenisation in buffers A/B, samples were centrifuged (12,000 rpm, 4 °C, 15 min). The resulting supernatant was divided: one part for protein quantification, the other neutralised to pH 7.0 (using kit-provided acid/base). Experiments were carried out according to the kit procedure and analysed using a multipurpose microplate reader. The absorbance of the samples in the 96-well plate was measured at 450 nm. The analysis kit was purchased from Suzhou Grace Biotechnology Co., Ltd (Cat. G0802W, Suzhou, China).

### 2.12. G6PDH, PKM and HK enzymatic activity measurement

Tissue samples were homogenised, centrifuged, and the supernatant was collected. For cell samples, cells were harvested into extraction buffer after sonication (200 W for 30 cycles with a power-off interval of 3s/10s), followed by centrifugation. Then the supernatant was used to calculate protein concentration and then was mixed with the corresponding assay reagent and absorbance was read with a multi-functional microplate reader. Absorbance was measured at 450 nm (G6PD, Cat. G0805W) and 340 nm (PKM, Cat. G0811W48; HK, Cat. G0810W). All kits were from Suzhou Grace Biotechnology Co., Ltd. (Suzhou, China) and used per the manufacturer's instructions.

### 2.13. Progesterone assay

Serum progesterone concentration was quantified using a commercial ELISA kit (Cat. HY-C006, Huaying Biotechnology, China) following the manufacturer’s protocol. The intra- and inter-assay coefficients of variation for progesterone were below 5% and 10%, respectively.

### 2.14. Cell viability assay

Cell viability was measured using Cell Counting Kit-8 (CCK-8, Abbkine, Cat. BMU106). Luteal primary cells were planted into 96-well plates at the density of 20,000 cells/well. After the cells were adhered, they were treated with drugs according to experimental needs. Then, the cells were incubated with CCK-8 reagent (diluted 1:10 (v/v) in culture medium) for 1 hour. Absorbance was read at 450 nm.

### 2.15. Lipid peroxidation measurement

Cells were seeded in six-well plates and treated with the indicated agents after attachment. Cells were then incubated with 5 μM C11-BODIPY 581/591 in fresh serum-free medium at 37 °C for 30 min in the dark. After trypsinization, the cells were washed three times with serum-free medium to remove unbound probe. Fluorescence was measured using a BD flow cytometer with the FITC channel, and the mean fluorescence intensity (MFI) was analysed using FlowJo software. C11-BODIPY was purchased from Dojindo Laboratories (Cat. L267).

### 2.16. Ferrous ion fluorescence staining

For cellular staining, cells were seeded in 24-well plates and treated with the indicated agents after attachment. After treatment, the cells were washed three times with PBS and incubated with 1 μM FerrOrange probe at 37 °C for 30 min in the dark. Fluorescence images were then acquired using an inverted fluorescence microscope. FerrOrange was purchased from Dojindo Laboratories (Cat. F374).

### 2.17. Targeted metabolomics analysis

Targeted metabolomics analysis of ovarian tissues was performed by Metware Biotechnology (Wuhan, China) with the AB Sciex QTRAP LC–MS/MS system.

### 2.18. Statistical analysis

Data are presented as mean ± SD. Statistical analyses were performed using SPSS 27.0 and GraphPad Prism 8.0 (8.3.1). Normality and homogeneity of variance were assessed by the Shapiro-Wilk test and Levene’s test, respectively. For positive-valued ratio or fold-change data, natural log transformation was applied where appropriate to improve distribution properties prior to parametric testing; figures display raw values for intuitive biological interpretation, while statistical calculations were based on transformed data when applicable. For two-group comparisons, an unpaired two-tailed Student’s t-test was used for normally distributed data with equal variance, Welch’s t-test for approximately normally distributed data with unequal variance, and the Mann–Whitney U test for data with marked deviation from normal distribution. For comparisons across three or more groups, the same distribution and variance criteria applied: one-way ANOVA followed by Tukey’s test for equal-variance normal data, Welch’s ANOVA with Games–Howell post hoc test for unequal-variance approximately normal data, and Kruskal–Wallis test with Dunn’s test for data with marked non-normality. All tests were two-sided, and *P* < 0.05 was considered statistically significant.

All in vitro experiments were performed with at least three independent biological replicates unless otherwise stated. Samples were randomly allocated to experimental groups and processed in parallel under identical culture conditions, reagent batches, and instrument settings. For objective instrumental quantitative assays, results are based on standardised instrument readouts and software quantification. Representative histological and immunohistochemical images were selected using consistent criteria of tissue integrity and staining quality applied uniformly across groups.

## 3. Results

### 3.1. Ferroptosis-related alterations occur in the postpartum and PGF2α-induced luteolysis models

**Figure 1.**
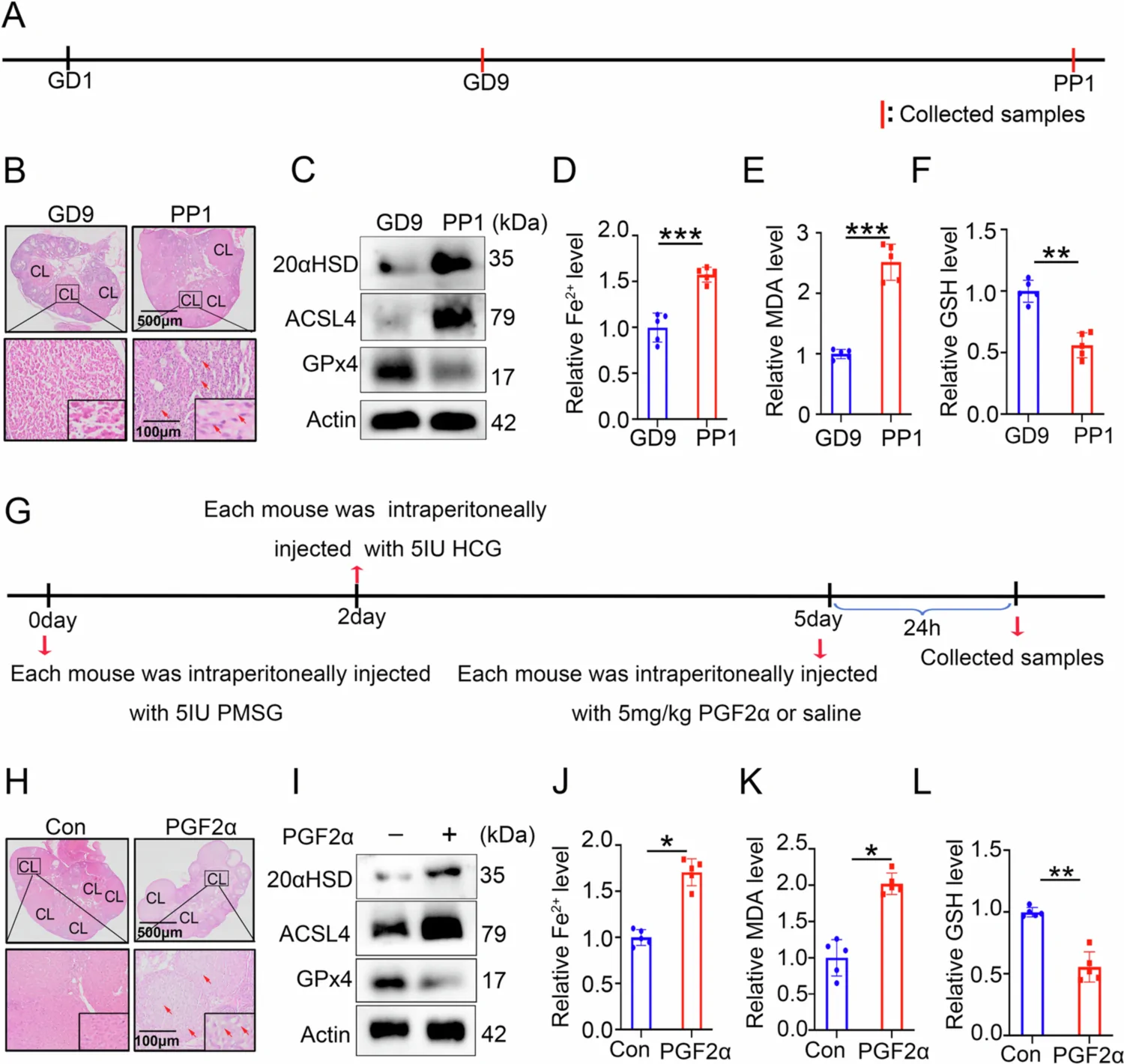
Ferroptosis is involved in the postpartum and PGF2α-induced luteolysis model. (A) The schematic diagram illustrates construction of postpartum luteolysis model and the collection of experimental samples on gestational day 9 (GD9) and postpartum day 1 (PP1). (B) Hematoxylin and eosin (HE) staining of ovaries from GD9 and PP1. Red arrows indicate vacuolisation. (C) Representative immunoblotting pictures of 20α-hydroxysteroid dehydrogenase (20αHSD) and ferroptosis marker in ovaries from GD9 and PP1. (D, E, F) Quantification of Fe^2+^ (D), malondialdehyde (MDA) (E) and glutathione (GSH) levels (F) in ovaries from GD9 and PP1. (G) The schematic timeline illustrates a prostaglandin F2α(PGF2α)-induced luteolysis model. (H) HE staining of ovaries from control and PGF2α-induced group. Red arrows indicate vacuolisation. (I) Representative immunoblotting pictures of 20αHSD and ferroptosis marker in ovaries from control and PGF2α-induced group. (J, K, L) Quantification of Fe^2^⁺ (J), MDA (K) and GSH levels in ovaries from control and PGF2α-induced group. The results shown in panels C–F and I–L were obtained from five independent biological replicates, and representative HE images in panels B and H are representative of three biological replicates. Biochemical data in D–F and J–L were normalized per‑sample to the mean of GD9 (D–F) or control (J–L), and shown as relative levels. Data are shown as mean ± SD. Welch’s t-test was used for panel E; unpaired two-tailed Student’s t-test was used for panels D, F, and J–L. *p < 0.05; **p < 0.01; ***p < 0.001.

To further substantiate the involvement of ferroptosis in the regression of the corpus luteum, we collected ovarian tissues from GD9 and PP1 (Figure 1A). Histological analyses revealed pronounced structural degeneration of corpora lutea on PP1, characterised by increased vacuolisation and reduced cell density in HE staining (Figure 1B). At the molecular level, the expression of the luteolysis marker gene 20αHSD was significantly upregulated on PP1 (Figure 1C). Furthermore, we observed marked signatures of ferroptosis in the PP1 group compared to GD9, including strongly elevated ACSL4 expression, substantially diminished GPx4 levels, increased Fe^2^⁺ and MDA accumulation, and a marked decrease in GSH content (Figure 1C−F). Consistently, immunohistochemical staining of ACSL4 and GPx4 showed the same expression patterns, while FerrOrange fluorescence staining revealed increased Fe^2^⁺ accumulation in PP1 ovarian tissues (Suppl.Fig1A, C).

In parallel, we employed a PGF2α-induced luteolysis model to experimentally assess ferroptosis (Figure 1G). Similar to the postpartum luteolysis model, HE staining showed increased vacuolisation and reduced cellular density in the PGF2α-induced group (Figure 1H). Moreover, these tissues exhibited elevated 20αHSD and ACSL4 expression, reduced GPx4 levels, a significant increase in Fe^2^⁺ and MDA content, and decreased GSH levels (Figure 1I−L), mirroring the molecular features observed during the postpartum luteolysis model. In agreement with these findings, immunohistochemical analysis confirmed enhanced ACSL4 staining and diminished GPx4 staining, while FerrOrange fluorescence demonstrated greater Fe^2^⁺ accumulation in ovarian tissues from the PGF2α-treated group (Suppl.Fig1B, D).

Taken together, these findings demonstrate prominent ferroptosis-related alterations during luteal regression.

### 3.2. 6-phosphogluconate accumulates and pyruvate levels decline in the postpartum luteolysis

**Figure 2.**
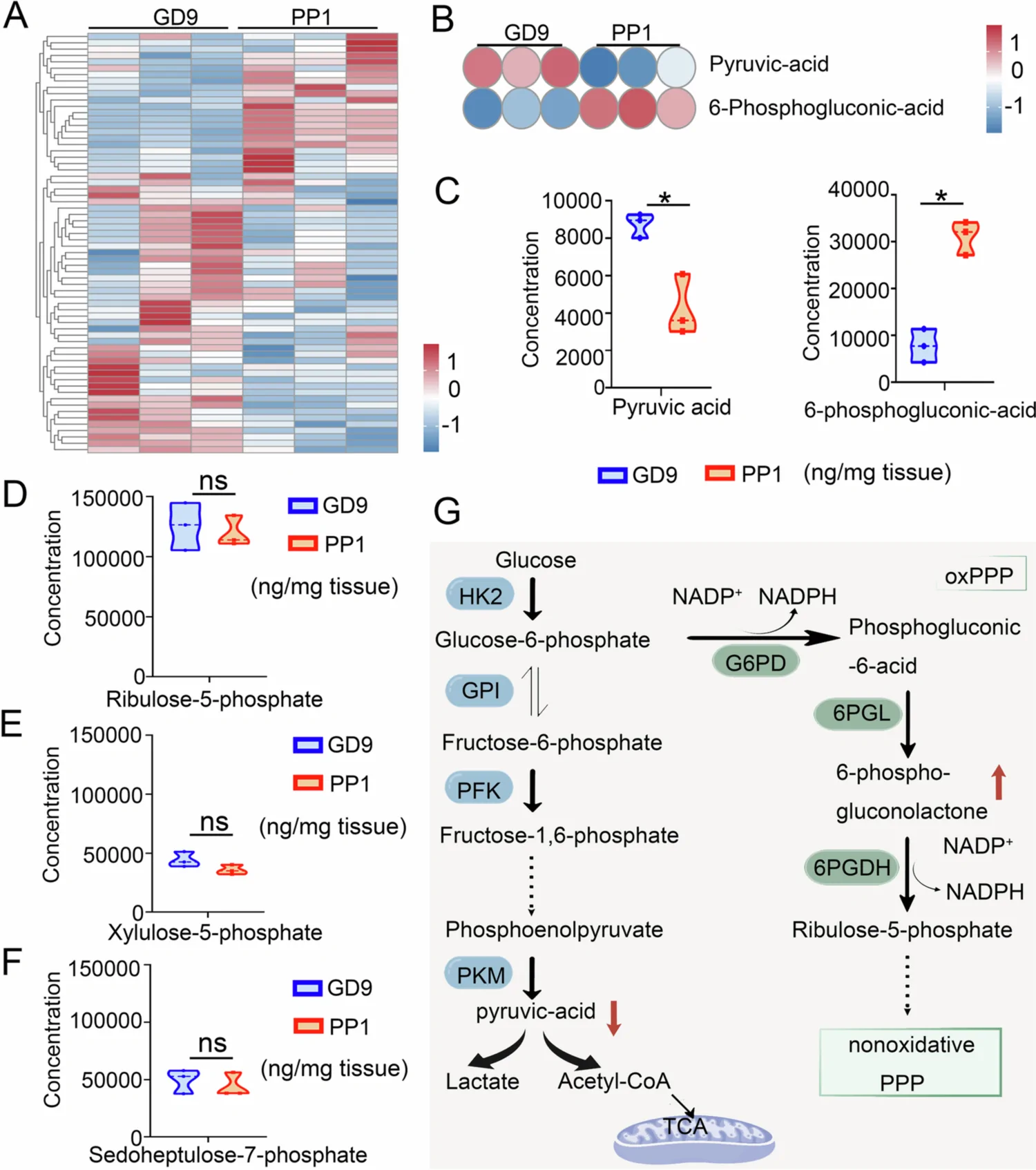
6-phosphogluconate accumulates and pyruvate levels decline in the postpartum luteolysis model. (A-B) Energy metabolism heatmap of luteal tissues from GD9 and PP1. (C) Relative concentrations of pyruvate and 6-phosphogluconic-acid on GD9 and PP1. (D-F) Relative concentrations of ribulose-5-phosphate (D), xylulose-5-phosphate levels (E) and sedoheptulose-7-phosphate (F) on GD9 and PP1. (G) Schematic diagram illustrating key metabolic pathways of glycolysis and the pentose phosphate pathway (PPP). The results shown in panels C–F were obtained from independent biological replicates. Data are shown as the mean ± SD. For statistical analysis, panel F was analysed using the Mann–Whitney U test, and panels C–E were analysed using the unpaired two-tailed Student’s t-test. ns, not significant (*p* > 0.05); *p < 0.05.

Given the metabolic dependence of ferroptosis and the high metabolic activity of the corpus luteum, we next performed targeted energy metabolomics on GD9 and PP1 ovarian tissues to determine whether luteal regression was accompanied by changes in energy metabolism. The data revealed a distinct metabolic shift during regression: the terminal glycolytic product pyruvate was similarly reduced, whereas the oxidative PPP intermediate 6-phosphogluconate accumulated noticeably on PP1 (Figure 2A−C). Interestingly, despite the accumulation of 6-phosphogluconate in the PPP, the downstream PPP metabolites, including ribulose-5-phosphate, sedoheptulose-7-phosphate, xylulose-5-phosphate remained unchanged on PP1 (Figure 2D−F), suggesting accumulation of an early oxidative PPP intermediate without detectable accumulation of downstream PPP intermediates.

Taken together, these results suggest energy metabolic remodelling involving the early oxidative PPP and reduced terminal glycolytic output (Figure 2G).

### 3.3. G6PD hyperactivation in the postpartum and PGF2α-induced luteolysis model

Metabolic remodelling is primarily reflected in the changes of key enzymes that participate in glucose metabolism. In order to clarify this, we made a quantitative analysis of the expression levels and activity of key enzymes for glucose metabolism in postpartum and PGF2α-induced luteolysis model.

**Figure 3.**
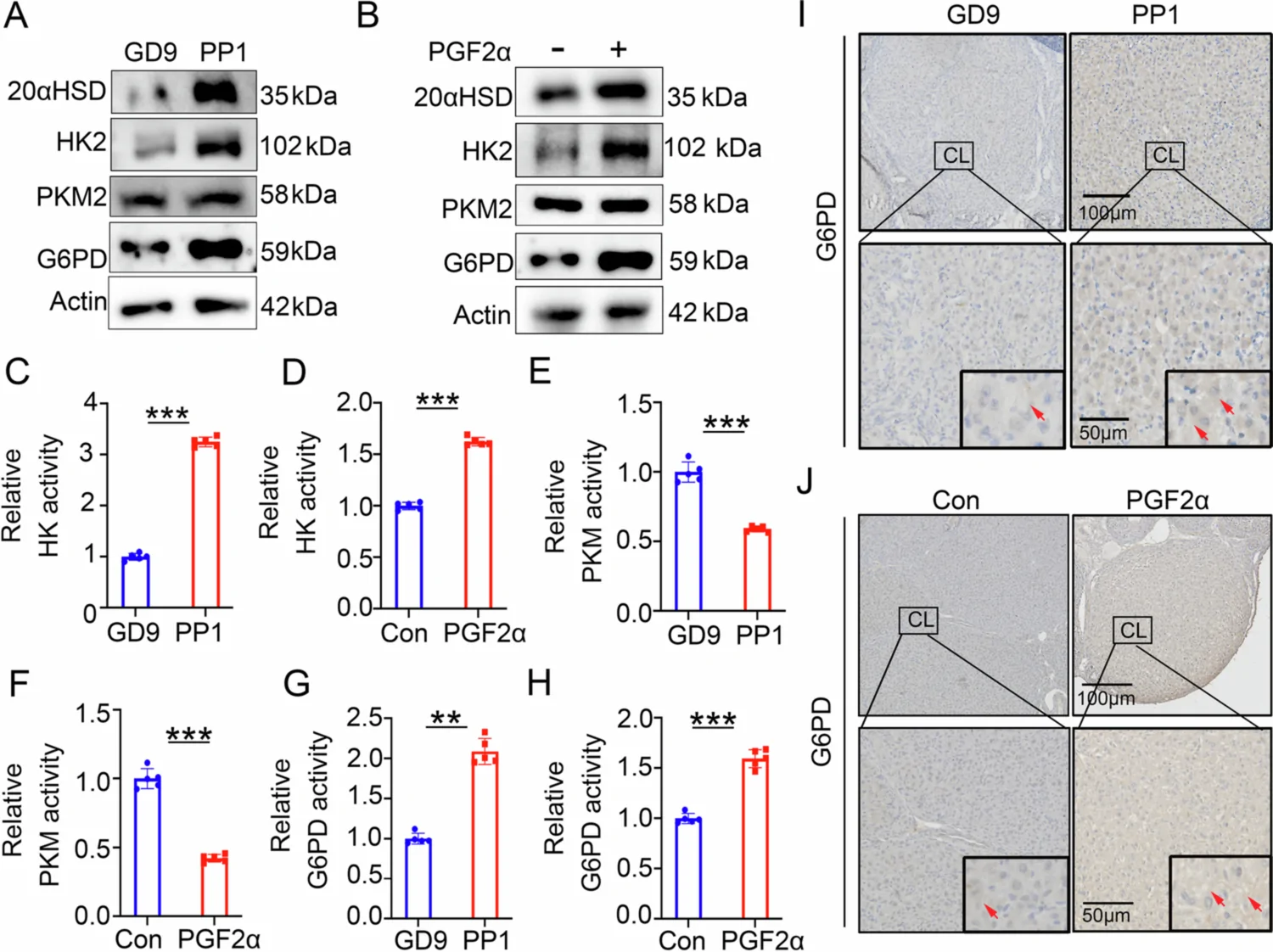
G6PD hyperactivation in the postpartum and PGF2α-induced luteolysis model. (A-B) Representative immunoblotting pictures of 20αHSD, Hexokinase 2(HK2), Pyruvate kinase M2(PKM2), and Glucose-6-phosphate dehydrogenase(G6PD) in ovaries from GD9 and PP1 (A), and control and PGF2α-induced groups (B). (C-H) Enzymatic activities of HK (C, D), PKM (E, F), and G6PD (G, H) were measured in ovarian tissue homogenates from GD9 and PP1 mice (C, E, G) and from control and PGF2α-induced mice (D, F, H). (I-J) Immunohistochemical analysis showing differential expression of G6PD in GD9 and PP1 (I) and control and PGF2α-induced group (J). Red arrows mark regions with positive signals. The results shown in panels A–H were obtained from five biological replicates, and representative IHC images in panels I–J are from three biological replicates. For enzymatic activity measurements in C–H, individual sample values were normalized to the mean of the GD9 group (C, E, G) or control group (D, F, H) and shown as relative levels. Data are shown as mean ± SD. Mann–Whitney U test was used for panel G; unpaired two-tailed Student’s t-test was used for panels C–F and H. **p < 0.01; ***p < 0.001.

Our findings showed that both HK2 protein abundance and HK activity were significantly increased in the PP1 (Figure 3A, C) and in the PGF2α-induced group (Figure 3B, D). In contrast, PKM2 protein abundance showed no obvious change, whereas PKM enzymatic activity was significantly decreased in both models (Figure 3A−B, E−F). This observation aligns with the reduction in pyruvate detected in our metabolomics analysis.

Notably, the levels of G6PD protein expression and enzymatic activity increased on PP1 (Figure 3A, G) and in the PGF2α-induced group (Figure 3B, H). Furthermore, Immunohistochemical analysis indicated that G6PD was highly expressed on PP1 (Figure 3I) and PGF2α-induced group (Figure 3J). Together, these findings indicate glucose metabolic remodelling during luteolysis, with G6PD-associated alterations in the oxidative PPP shared by both models.

### 3.4. NADPH depletion and redox imbalance in the postpartum and PGF2α-induced luteolysis model

**Figure 4.**
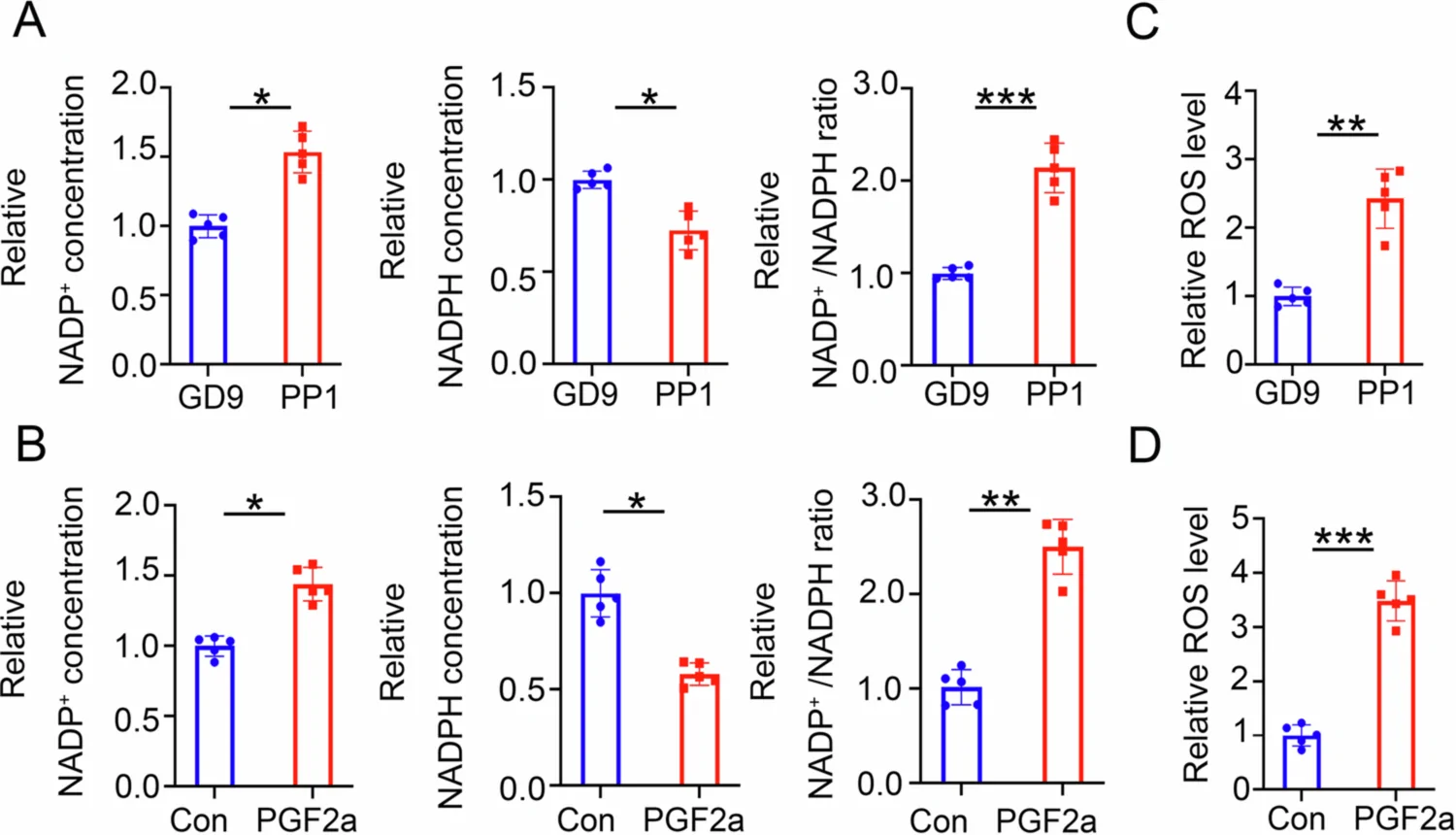
NADPH exhaustion and redox imbalance in the postpartum and PGF2α-induced luteolysis model. (A, B) Nicotinamide adenine dinucleotide phosphate (oxidised form, NADP⁺), Nicotinamide adenine dinucleotide phosphate (reduced form, NADPH), and the NADP⁺/NADPH ratio were measured in ovaries from GD9 and PP1 mice (A) and from control and PGF2α‑induced groups (B). (C, D) Reactive oxygen species (ROS) levels in ovarian tissue homogenates were quantified using a tissue ROS assay kit, as described in Section 2.9, in ovaries from GD9 and PP1 mice (C) and from control and PGF2α-induced groups (D). The results shown in panels A–D were obtained from five biological replicates. For measurements in A–D, individual sample values were normalized to the mean of GD9 (A, C) or control (B, D) and shown as relative levels. Data are shown as mean ± SD. For panel A, the NADP⁺/NADPH ratio was analysed using Welch’s t-test, whereas NADP⁺ and NADPH concentrations were analysed using the unpaired two-tailed Student’s t-test. Panels B–D were analysed using the unpaired two-tailed Student’s t-test. *p < 0.05; **p < 0.01; ***p < 0.001.

To determine whether increased G6PD expression and activity were accompanied by changes in the NADPH-related redox state during luteolysis, we measured NADPH, NADP⁺, and the NADP⁺/NADPH ratio in both the postpartum and PGF2α-induced luteolysis models. We found that, compared to GD9 and control groups respectively, NADPH levels were significantly lower in both the PP1 and PGF2α-induced groups, whereas NADP⁺ levels and the NADP⁺/NADPH ratio were markedly increased (Figure 4A−B). Furthermore, ROS levels in ovarian tissue homogenates were quantified using a tissue ROS assay kit, as described in Section 2.9. ROS levels were higher in the PP1 group than in the GD9 group (Figure 4C) and in the PGF2α-induced group than in the control group (Figure 4D).

Taken together, these data indicate a redox-imbalanced state during luteolysis, characterised by NADPH depletion and an increased NADP⁺/NADPH ratio despite elevated G6PD activity. The reduced steady-state NADPH level may reflect an imbalance between NADPH production and consumption through multiple NADPH-dependent metabolic and redox-regulating processes.

### 3.5. Upregulation of NOX2/NOX4 in the postpartum and PGF2α-induced luteolysis model

Given the concurrent increase in ROS and depletion of NADPH during luteolysis, together with the established role of NOX enzymes as NADPH-dependent sources of ROS, we next profiled the expression of NOX family members in ovarian tissues to identify isoforms potentially associated with this process.

**Figure 5.**
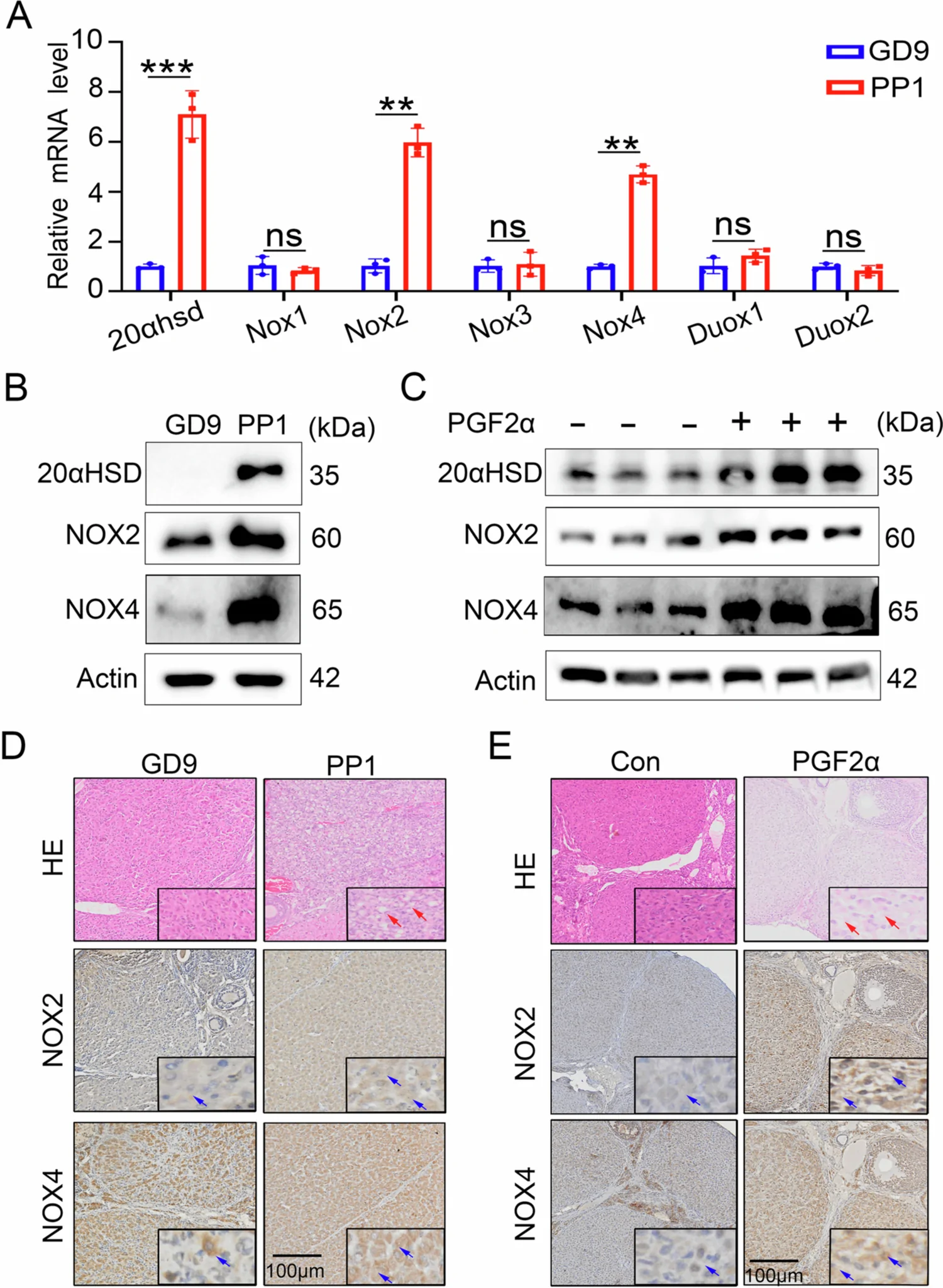
Upregulation of NOX2/NOX4 in the postpartum and PGF2α-induced luteolysis model. (A) Transcriptomic profiling of NADPH oxidase (NOX) family in the postpartum luteolysis model (GD9 vs. PP1). (B, C) Representative immunoblotting pictures of 20αHSD, NOX2 and NOX4 in ovaries from GD9 and PP1 (B), and control and PGF2α-induced groups (C). (D, E) IHC staining of NOX2 and NOX4 in ovarian sections from GD9 and PP1 (D) and control and PGF2α-induced (E) mice. Red arrows indicate vacuolisation and blue arrows mark regions with positive signals. The results shown in panels A–E were obtained from three independent biological replicates. Data are shown as mean ± SD. Panel A was analysed using an unpaired two-tailed Student’s t-test. ns, not significant (*p* > 0.05); **p < 0.01; ***p < 0.001.

To address this, we systematically analysed NOX expression. For the first time, the real-time quantitative PCR results showed that mRNA levels of Nox2 and Nox4 were significantly elevated on PP1, whereas other NOX family members (Nox1, Nox3, Duox1/2) remained largely unchanged (Figure 5A).

To confirm these observations, we perform western blotting analysis of NOX2 and NOX4 protein expression levels in the postpartum luteolysis model and the PGF2α-induced luteolysis model. Consistent with the transcriptomic data, the expression of NOX2 and NOX4 proteins was marked increased in the PP1 group (Figure 5B) and the PGF2α-induced group (Figure 5C). Further analysis by immunohistochemistry also demonstrated that NOX2 and NOX4 expression was substantially elevated in the PP1 group (Figure 5D) and PGF2α-induced group (Figure 5E), suggesting their active involvement in luteolysis.

### 3.6. PGF2α regulates NOX2/NOX4 expression in luteal cells

**Figure 6.**
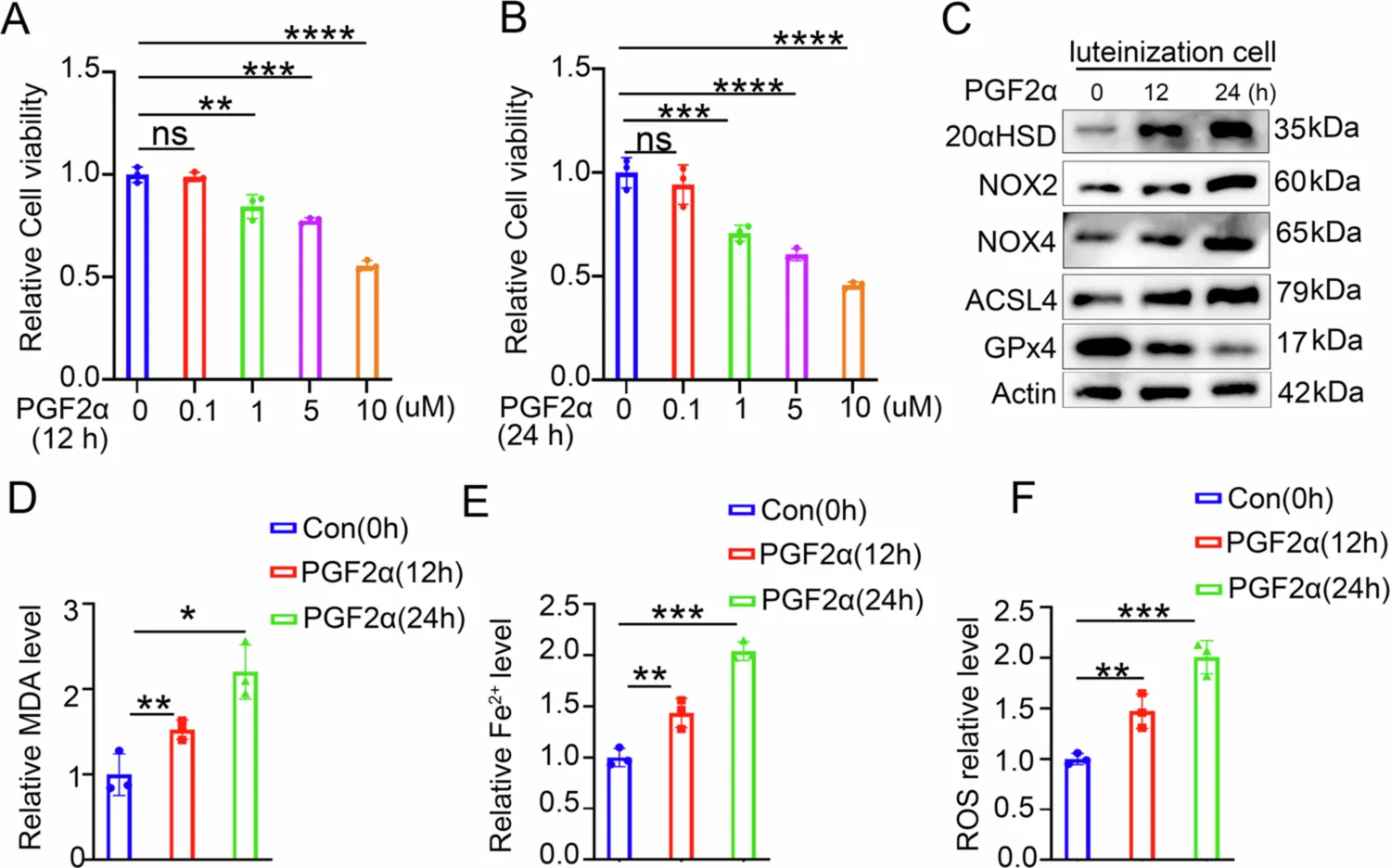
PGF2α regulates NOX2/NOX4 expression in luteal cells. (A-B) The viability of luteal cells treated with different concentrations of PGF2α was assessed at both 12 h (A) and 24 h (B) time points. (C) Primary luteal cells were stimulated with 1 μM PGF2α for either 12 or 24 hours to establish a time gradient, followed by detection of 20αHSD, NOX2, NOX4, ACSL4 and GPx4 protein expression. (D, E, F) MDA, Fe^2+^ and ROS levels were measured under control, PGF2α(12 h) and PGF2α(24 h) conditions. The results shown in panels A–F were obtained from three independent biological replicates. For measurements in A−B and D−F, individual sample values were normalized to the mean of the 0 h control group and shown as relative levels. Data are shown as mean ± SD. Panels A-B and D-F were analysed using the one-way ANOVA followed by Tukey’s post hoc test. ns, not significant (*p* > 0.05); *p < 0.05; **p < 0.01; ***p < 0.001; ****p < 0.0001.

To further determine whether PGF2α regulates NOX2 and NOX4 expression, we subsequently examined their expression in primary luteal cells treated with PGF2α. Primary luteal cells were isolated after induction of the corpus luteum and their identity was confirmed by measurement of the expression of genes encoding markers of luteal development (STAR and 3β-HSD; Suppl.Fig2A-C). To determine the optimal concentration of PGF2α for subsequent cellular assays, we first assessed its effects on cell viability across a range of doses (Figure 6A−B). Based on these results, concentrations of 1, 5, and 10 μM effectively inhibited cell viability. Consequently, 1 μM was selected for further experiments as it represented the lowest effective dose that balanced efficacy with minimal potential for cytotoxicity. Treatment with 1 μM PGF2α induced NOX2 and NOX4 expression in a time-dependent manner (Figure 6C). Furthermore, PGF2α induced ferroptotic characteristics of luteal cells in a time-dependent manner, primarily manifested by increased ACSL4 expression, decreased GPx4 levels, and significant accumulation of MDA and Fe^2+^ content (Figure 6C−E). Consistently, FerrOrange fluorescence staining revealed a time-dependent increase in intracellular Fe^2^⁺ levels following PGF2α treatment (Suppl.Fig2D). Moreover, ROS levels were also significantly upregulated (Figure 6F).

Together, these findings show that PGF2α time-dependently increases NOX2/NOX4 expression, ROS accumulation, and ferroptosis-related alterations in luteal cells.

### 3.7. Knockdown of NOX2/NOX4 attenuates G6PD expression and ferroptosis during PGF2α-induced luteolysis

**Figure 7.**
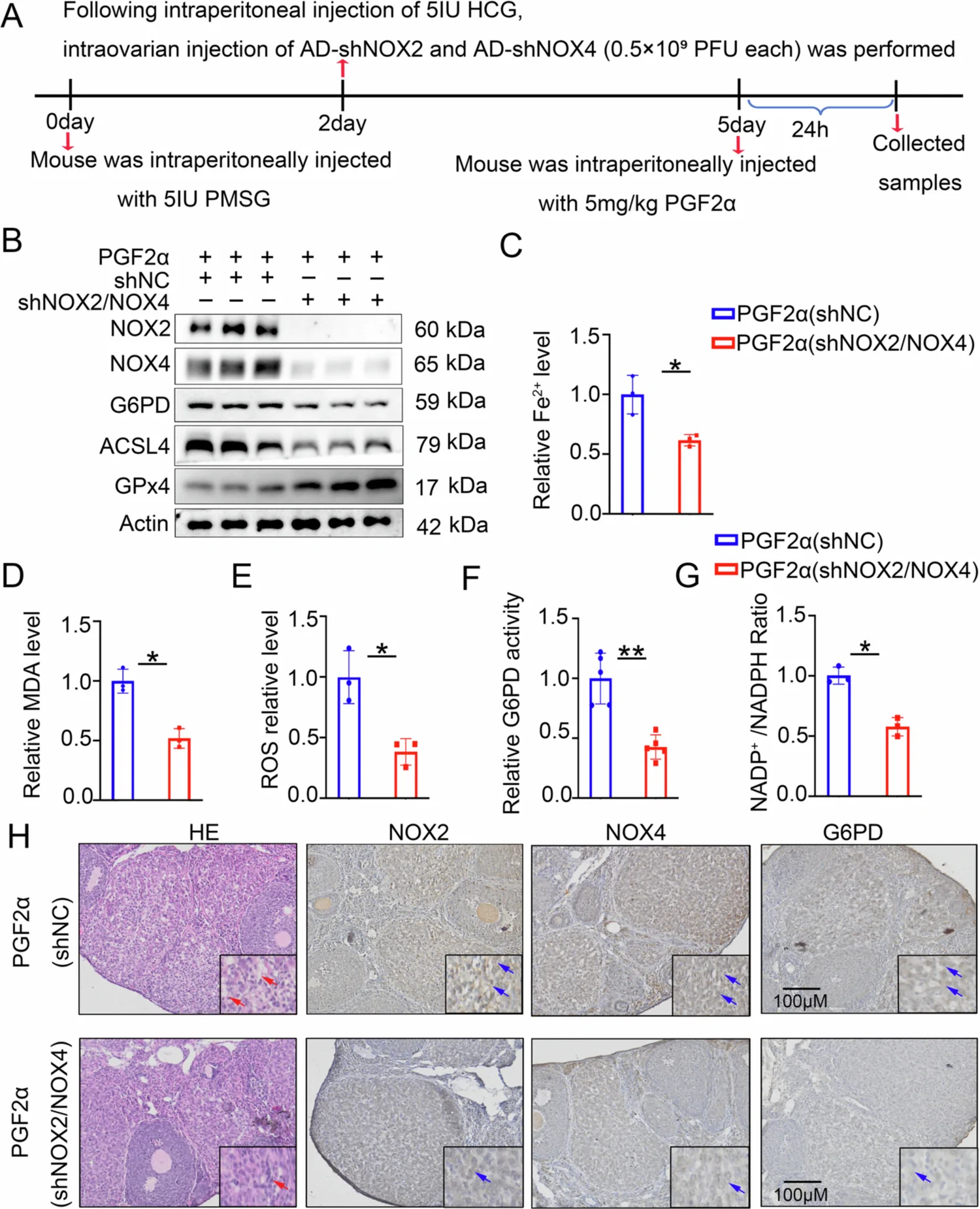
Knockdown of NOX2/NOX4 attenuates G6PD expression and ferroptosis during the PGF2α-induced luteolysis model. (A) Schematic diagram of in situ ovarian injection of adenoviruses (AD-shNOX2/shNOX4) for the PGF2α-induced luteolysis model. (B) Representative immunoblotting pictures of NOX2, NOX4, G6PD, Acyl-CoA synthetase long-chain family member 4 (ACSL4) and Glutathione peroxidase 4 (GPx4) in PGF2α-induced luteolysis model of AD-shNC versus AD-shNOX2/shNOX4 double-knockdown. (C-E) Fe^2^⁺, MDA, and ROS levels in luteal tissues from the PGF2α-induced luteolysis model: AD-shNC versus AD-shNOX2/shNOX4 groups. (F) G6PD enzymatic activity in luteal tissues following NOX2/NOX4 knockdown. (G) NADP^+^/NADPH ratio in luteal tissues from the PGF2α-induced luteolysis model: AD-shNC versus AD-shNOX2/shNOX4 groups. (H) HE staining and IHC detection of NOX2, NOX4 and G6PD expression in luteal sections within the PGF2α-induced luteolysis model, comparing AD-shNC and AD-shNOX2/shNOX4 groups. Red arrows indicate vacuolisation and blue arrows mark regions with positive signals. The results shown in panel F was obtained from five independent biological replicates, while those shown in panels B–E and G-H were obtained from three independent biological replicates. For measurements in C‑G, individual sample values were normalized to the mean of the PGF2α(shNC) group and shown as relative levels. Data are shown as mean ± SD. Panels C-D and G were analysed using the unpaired two-tailed Student’s t-test. Panels E was performed on natural-log-transformed values using an unpaired two-tailed Student’s t-test. Welch’s t-test was used for panel F. *p < 0.05; **p < 0.01.

To further elucidate the roles of NOX2 and NOX4 in luteolysis, we performed combined NOX2/NOX4 knockdown experiments. To this end, and based on the observed upregulation of NOX2/NOX4 by PGF2α, we utilised a superovulation-induced luteal model. Ovaries of 21-day-old superovulated mice were injected in situ with control adenovirus or two shRNA adenoviruses separately targeting NOX2 and NOX4 for combined knockdown, followed by PGF2α-induced luteolysis (Figure 7A). Tissues were subsequently harvested to assess protein levels of key regulators. Efficient knockdown of NOX2 and NOX4 was confirmed in luteal tissues (Figure 7B). Importantly, combined knockdown of NOX2/NOX4 led to changes in ferroptosis-related markers: ACSL4 expression decreased, while GPx4 levels increased. Correspondingly, Fe^2^⁺ content, MDA concentrations, and ROS accumulation were all markedly reduced (Figure 7C−E). Notably, NOX2/NOX4 knockdown reduced G6PD protein expression and enzymatic activity and attenuated the PGF2α-induced elevation of the NADP⁺/NADPH ratio (Figure 7B, F−G). Histopathological analysis and IHC staining further verified these results: silencing NOX2/NOX4 preserved luteal structure, delayed luteal regression, and restored G6PD expression patterns toward normal levels (Figure 7H). Regarding glucose metabolism, a remarkable increase of PKM enzymatic activity was found after silencing the expression of NOX2/NOX4 (Suppl. Fig. 3 A), whereas lactate and pyruvate levels remained unchanged (Suppl. Fig. 3B−C).

Taken together, these results show that combined NOX2/NOX4 knockdown attenuates PGF2α-induced oxidative stress, G6PD activation, and ferroptosis.

### 3.8. Inhibiting NOX2/NOX4 or ferroptosis suppresses PGF2α-induced G6PD hyperactivation in luteal cells

To further elucidate the interplay between aberrant G6PD activation and NOX2/NOX4 expression, ferroptosis, and oxidative stress, we performed a series of experiments in primary luteal cells to examine G6PD expression and enzymatic activity under PGF2α modulated conditions.

**Figure 8.**
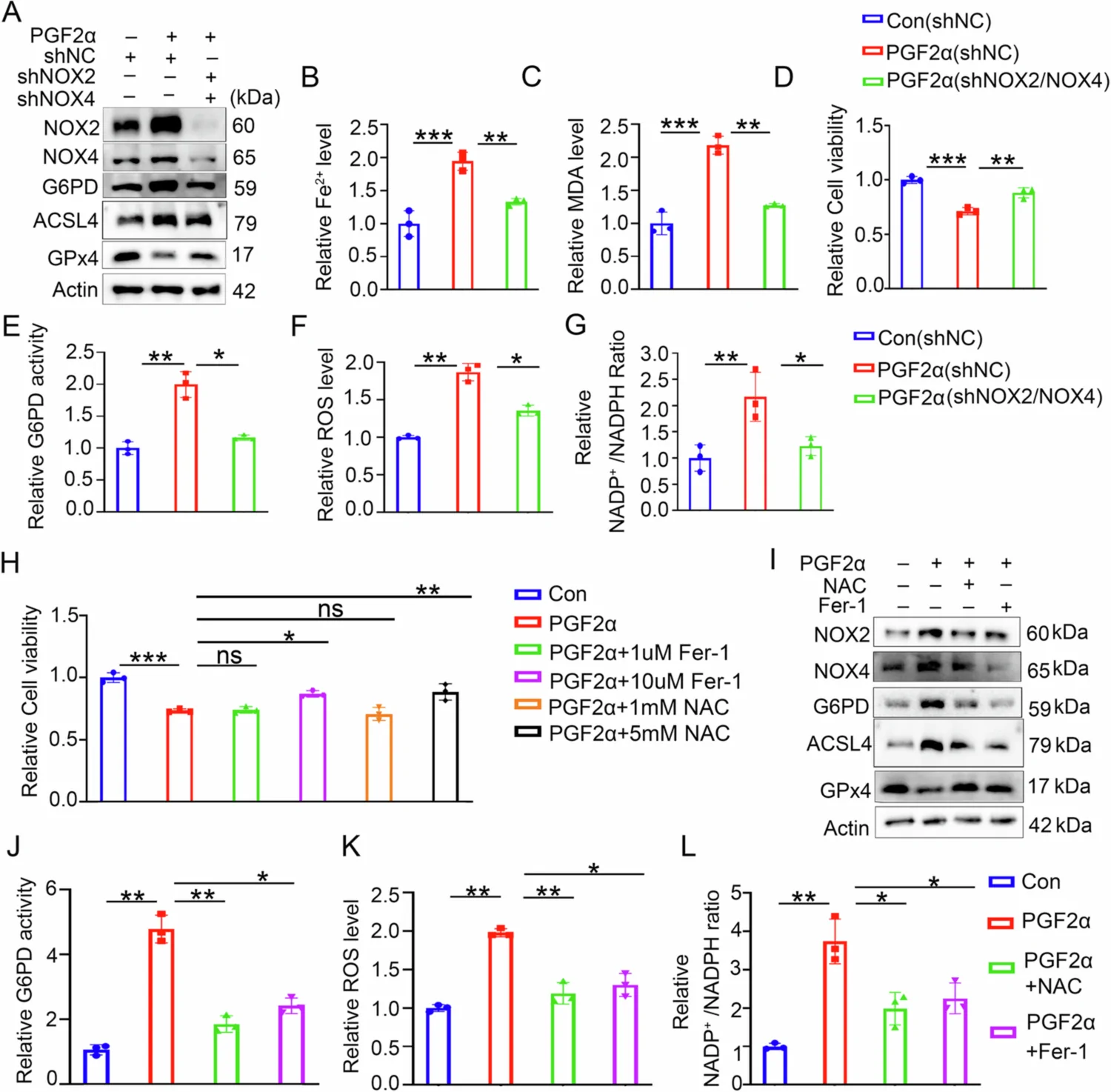
Inhibiting NOX2/NOX4 or ferroptosis suppresses PGF2α-Induced G6PD hyperactivation in luteal Cells. (A) Representative immunoblotting pictures of G6PD, NOX2, NOX4, ACSL4 and GPx4 expression in primary luteal cells, under control, PGF2α, and PGF2α + shNOX2/shNOX4 conditions. (B, C) Quantification of Fe^2+^ levels (B)and MDA levels (C) in the above groups. (D) The analysis of 1 μM PGF2α-induced luteal cell viability in the above groups. (E, F, G) Quantification of G6PD enzymatic activity (E), ROS levels (F), and the NADP⁺/NADPH ratio (G) in the above groups. (H) The analysis of PGF2α-induced luteal cell viability treated with different concentrations of N-acetylcysteine (NAC) or Ferrostatin-1(Fer-1). (I) Representative immunoblotting pictures of G6PD, NOX2, NOX4, ACSL4 and GPx4 expression in primary luteal cells, under control, PGF2α, PGF2α + NAC and PGF2α + Fer-1 conditions. (J, K, L) Quantification of G6PD enzymatic activity (J), ROS levels (K), and the NADP⁺/NADPH ratio (L) in the above groups. Cells were treated with 1 μM PGF2α for 4 hours. Following co-treatment with shNC or shNOX2/NOX4 adenovirus (MOI = 60) for 48 hours, samples were collected for analysis. Cells were treated with 1 μM PGF2α for 4 hours. This was followed by a 20-hour co-treatment with 10 μM Fer-1 or 5 mM NAC. Samples were then harvested for analysis. The results shown in panels A–L were obtained from three independent biological replicates. For measurements in B‑G and H, J–L, individual sample values were normalized to the mean of Con(shNC) (B–G) or Con (H, J–L) and shown as relative levels. Data are shown as mean ± SD. Panels B–D, G–H, and J–L were analysed using the one-way ANOVA followed by Tukey’s post hoc test. Panels E was performed on natural-log-transformed values using an unpaired two-tailed Student’s t-test. Welch’s t-test was used for panel F. ns, not significant (*p* > 0.05); *p < 0.05; **p < 0.01; ***p < 0.001.

First, NOX2 and NOX4 were simultaneously knocked down via adenoviral shRNA transduction. The expression of NOX2/NOX4 was markedly reduced at 48 hours post‑viral infection (Figure 8A). In PGF2α-induced oxidative stress models, combined NOX2/NOX4 knockdown attenuated ferroptosis-related alterations, as evidenced by decreased ACSL4 protein expression, increased GPx4 levels, and reduced Fe^2+^and MDA content (Figure 8A−C). Consistently, FerrOrange fluorescence staining demonstrated reduced intracellular Fe^2^⁺ accumulation, while C11-BODIPY staining showed decreased lipid peroxidation following NOX2/NOX4 knockdown (Suppl. Fig. 4A-B). Furthermore, compared with PGF2α treatment alone, combined NOX2/NOX4 knockdown decreased G6PD protein expression and enzymatic activity (Figure 8A,E), increased cell viability (Figure 8D), and reduced ROS levels (Figure 8F) and lowered the cellular NADP⁺/NADPH ratio (Figure 8G). These findings are consistent with the *in vivo* results and suggest an association between NOX2/NOX4 and PGF2α-induced G6PD activation in luteal cells.

To better illustrate the regulatory relationship between oxidative stress and G6PD, we further treated cells with NAC, a scavenger of ROS. The data showed that 5 mM NAC significantly restored the cell viability impaired by PGF2α (Figure 8H). 5 mM NAC treatment markedly reduced G6PD expression and activity (Figure 8I−J), and reduced cellular levels of ROS (Figure 8K). We next used Fer-1 to determine whether suppression of ferroptosis-related lipid peroxidative stress affected PGF2α-induced G6PD activation. Similarly, 10 μM Fer-1 effectively restored the PGF2α-inhibited cell viability (Figure 8H). 10 μM Fer-1 treatment suppressed G6PD expression and activity (Figure 8I−J) and reduced cellular levels of ROS (Figure 8K). A reduction in the NADP⁺/NADPH ratio was also observed in cells co-treated with either NAC or Fer-1, compared to those treated with PGF2α alone (Figure 8L). Notably, treatment with either NAC or Fer-1 suppressed the PGF2α-induced upregulation of NOX2 and NOX4 expression (Figure 8I).

Taken together, reducing oxidative stress attenuates PGF2α-induced G6PD activation in luteal cells.

### 3.9. Pharmacological and genetic suppression of G6PD attenuates PGF2α-induced ferroptosis in primary luteal cells

**Figure 9.**
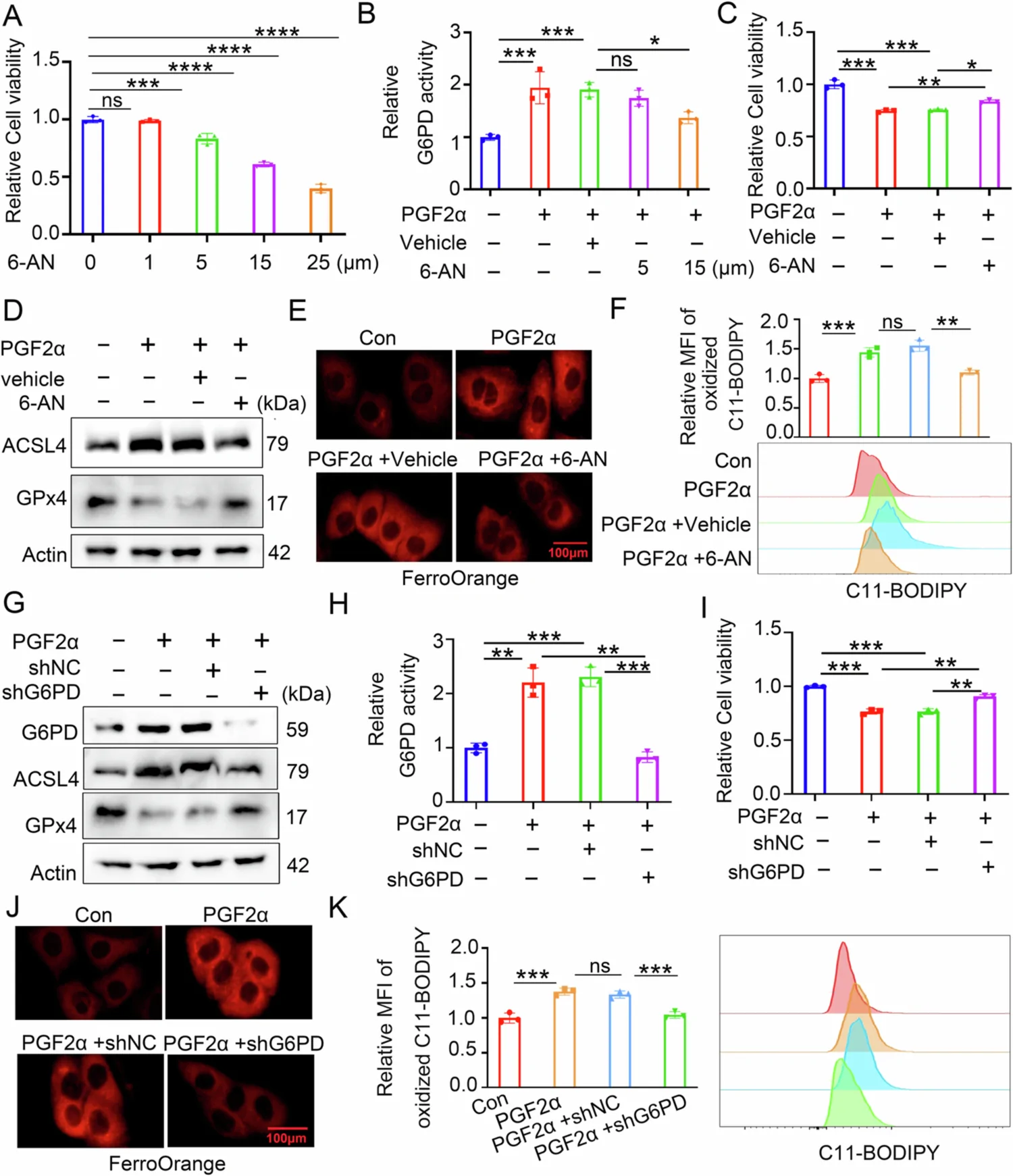
Pharmacological and genetic suppression of G6PD attenuates PGF2α-induced ferroptosis in primary luteal cells. (A) Cell viability of primary luteal cells treated with 6-AN at 0, 1, 5, 15, or 25 μM. (B) G6PD activity in control and PGF2α-treated cells exposed to vehicle, 5 μM or 15 μM 6-AN. (C) Cell viability in control and PGF2α-treated cells with or without 15 μM 6-AN. (D) Representative western blots of ACSL4 and GPX4 in the indicated groups. (E) Representative FerrOrange fluorescence images of intracellular Fe^2^⁺. (F) Representative flow-cytometric profiles and relative mean fluorescence intensity (MFI) quantification of oxidised C11-BODIPY for lipid peroxidation assessment. Cells were co-treated with 1 μM PGF2α and 15 μM 6-AN for 24 h, except that C11-BODIPY analysis was performed after 12 h. (G) Representative western blots of G6PD, ACSL4, and GPX4 in control and PGF2α-treated cells transduced with Ad-shNC or Ad-shG6PD. (H) G6PD activity in the indicated groups. (I) Cell viability in the indicated groups. (J) Representative FerrOrange fluorescence images of intracellular Fe^2^⁺. For the above experiments, cells were treated with 1 μM PGF2α for 4 hours. Following co-treatment with shNC or shG6PD adenovirus (MOI = 60) for 48 hours. (K) Representative flow-cytometric profiles and relative MFI quantification of oxidised C11-BODIPY for lipid peroxidation assessment. Cells were transduced with Ad-shNC or Ad-shG6PD for 24 h, followed by 1 μM PGF2α treatment for 12 h before C11-BODIPY analysis. For measurements in A–C, F, H–I, K, individual sample values were normalized to the mean of the 0 μM vehicle control (A–C, F) or shNC control (H–I, K) and shown as relative levels. Data are presented as mean ± SD. Statistical analyses for panels A–C, F, H–I, and K were performed using one-way ANOVA followed by Tukey’s post hoc test. ns, not significant; *p < 0.05, **p < 0.01, ***p < 0.001, and ****p < 0.0001. (*n* = 3 independent biological experiments).

To determine whether G6PD activation is functionally involved in PGF2α-induced ferroptosis, we first investigated its role at the cellular level. Dose–response analysis showed that 6-AN reduced primary luteal cell viability at relatively high concentrations, whereas 15 μM effectively inhibited PGF2α-induced G6PD activation without causing marked basal cytotoxicity (Figure 9A−B). Therefore, 15 μM 6-AN was selected for subsequent experiments. Pharmacological inhibition of G6PD with 6-AN alleviated PGF2α-induced cell viability impairment, as well as the suppression of GPX4 and elevation of ACSL4 expression (Figure 9C−D). In parallel, FerrOrange fluorescence staining and C11-BODIPY analysis demonstrated that 6-AN attenuated intracellular Fe^2^⁺ accumulation and lipid peroxidation induced by PGF2α (Figure 9E−F). To further validate these findings genetically, G6PD was knocked down using Ad-shG6PD. G6PD knockdown reduced G6PD enzymatic activity, restored cell viability, normalised ACSL4 and GPX4 expression, and alleviated PGF2α-induced Fe^2^⁺ accumulation and lipid peroxidation (Figure 9G−K). Collectively, these cellular findings indicate that G6PD activation contributes to PGF2α-induced ferroptosis in luteal cells.

### 3.10. Pharmacological inhibition of G6PD attenuates ferroptosis and partially restores luteal steroidogenic function in PGF2α-induced luteolysis

**Figure 10.**
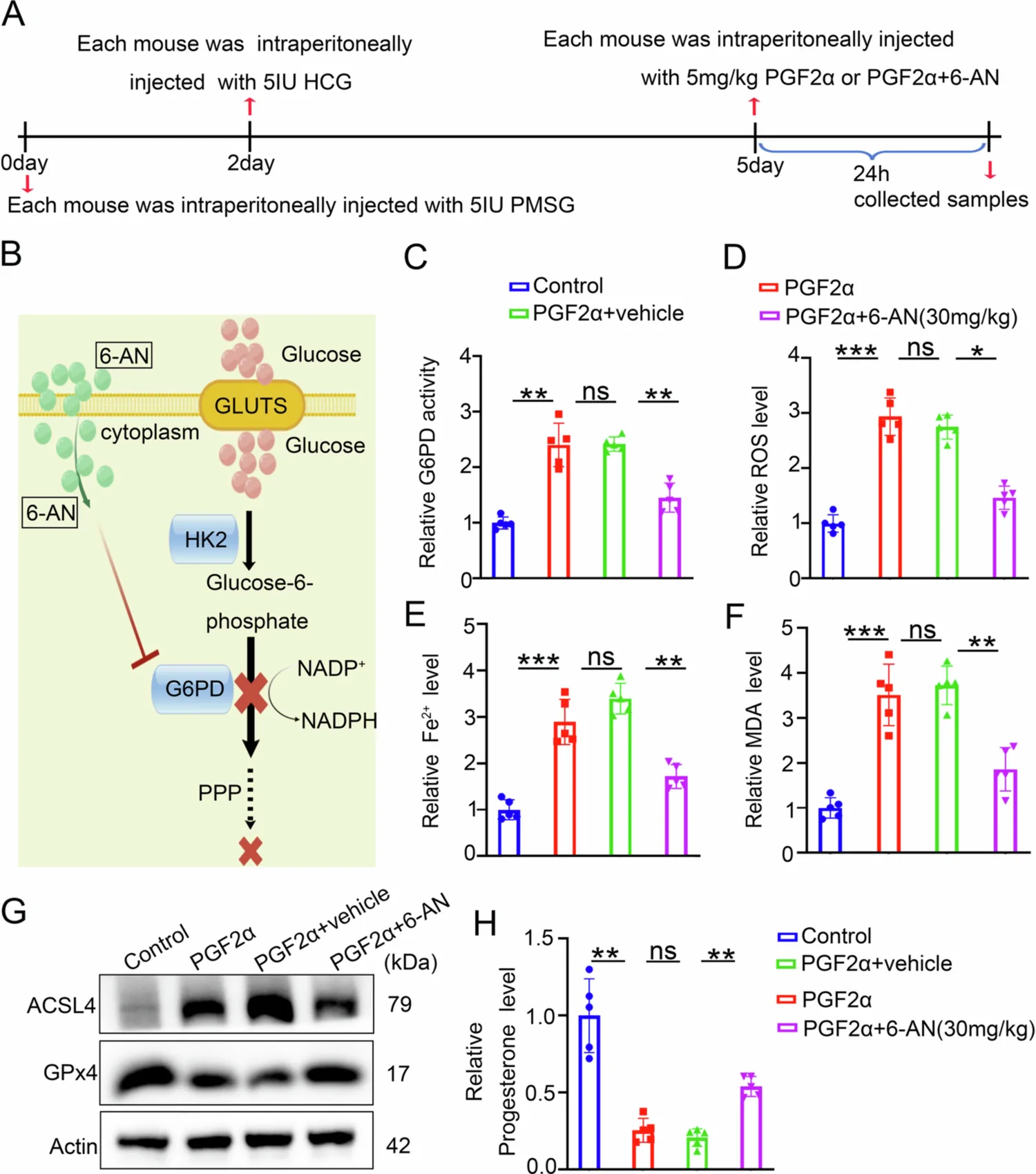
Pharmacological inhibition of G6PD attenuates ferroptosis and partially restores luteal steroidogenic function in PGF2α-induced luteolysis. (A) The schematic timeline outlines the key steps in establishing the PGF2α-induced luteolysis model, including treatment with 6-Aminonicotinamide (6-AN). (B) Illustration of the mechanism and molecular targets of 6-AN. (C, D, E, F) G6PD activity (C), ROS levels (D), Fe^2+^ levels (E) and MDA levels (F) was measured under control, PGF2α, PGF2α + vehicle, and PGF2α + 6-AN conditions. (G) Ferroptosis-related markers (ACSL4 and GPx4) protein levels under control, PGF2α, PGF2α + vehicle, and PGF2α + 6-AN conditions. (H) Progesterone levels under control, PGF2α, PGF2α + vehicle, and PGF2α + 6-AN conditions. The results shown in panels C–H were obtained from five independent biological replicates. For measurements in C–F, H, individual sample values were normalized to the mean of the Control group and shown as relative levels. Data are shown as mean ± SD. One-way ANOVA followed by Tukey’s post hoc test was used for Panels C–F. Welch’s ANOVA + Games–Howell post hoc test was used for panel H. ns, not significant (*p* > 0.05); *p < 0.05; **p < 0.01; ***p < 0.001.

Building on these cellular findings, we next examined whether 6-AN treatment could similarly alleviate ferroptosis and luteal dysfunction *in vivo*. To this end, we employed the G6PD inhibitor 6-AN in the PGF2α-induced luteolysis model (Figure 10A−B). To determine the appropriate *in vivo* concentration of 6-AN for inhibiting G6PD, mice were intraperitoneally injected with a dose range of 0, 5, 10, 30, and 50 mg/kg in PGF2α-induced models. Analysis of G6PD enzymatic activity revealed that both 30 and 50 mg/kg doses significantly suppressed G6PD function (Suppl. Fig. 5A-C). Based on the principle of using the minimal effective dose to mitigate potential off-target or toxic effects, 30 mg/kg was selected for total subsequent experiments. As shown in Figure 10C, 6-AN effectively inhibited the enzymatic activity of G6PD during PGF2α-induced luteolysis. In subsequent experiments, we examined the effects of decreased G6PD enzymatic activity induced by 6-AN on ferroptosis levels and luteolysis status during luteolysis. We found that the levels of ROS, Fe^2^⁺ and MDA are significantly elevated in the PGF2α-induced luteolysis model, while co-treatment with 6-AN can alleviate the PGF2α-induced increases in ROS, Fe^2^⁺ and MDA levels (Figure 10D−F). Moreover, the key ferroptosis protein ACSL4 was significantly downregulated, while GPx4 protein was markedly upregulated after 6-AN treatment during PGF2α-induced luteolysis (Figure 10G). These results indicate that 6-AN treatment attenuated oxidative stress and ferroptosis during PGF2α-induced luteolysis. Furthermore, to further clarify the functional role of G6PD, we detected the progesterone content secreted by the corpus luteum. The results showed that PGF2α significantly reduced progesterone levels (Figure 10H). However, compared to the PGF2α model group, the 6-AN co-treatment group showed increased progesterone content (Figure 10H).

Collectively, G6PD inhibition attenuated PGF2α-induced ferroptosis-related alterations and partially preserved luteal steroidogenic function.

## 4. Discussion

Although our previous work established the involvement of ferroptosis in corpus luteum regression [16], the upstream oxidative signals and their relationship with metabolic remodelling remained unclear. Here, we report three principal findings. First, luteolysis is accompanied by distinct metabolic remodelling, with G6PD hyperactivation but NADPH depletion. Second, PGF2α upregulates NOX2 and NOX4, and combined NOX2/NOX4 knockdown attenuates oxidative stress and ferroptosis during luteal regression. Third, under high oxidative stress conditions, aberrantly activated G6PD appears to contribute to, rather than protect against, ferroptosis and luteolysis progression. Collectively, these findings support a model in which NOX2/NOX4-associated oxidative stress contributes to aberrant G6PD activation, redox imbalance, and ferroptosis during luteal regression.

The physiological postpartum luteolysis model based on GD9 and PP1 samples provides a context in which the corpus luteum has irreversibly committed to regression [1,40,41]. We used this model to identify metabolism-related changes under physiological conditions, and employed the PGF2α-induced luteolysis model as a temporally controllable system to validate and mechanistically investigate these findings. Metabolomic and enzymatic analyses revealed a coordinated shift in glucose metabolism during luteal regression, characterised by reduced terminal glycolytic output (decreased pyruvate and PKM activity) and increased involvement of the early oxidative PPP, as indicated by elevated G6PD protein expression, G6PD activity, and 6-phosphogluconate accumulation. An increase in HK2 protein abundance, accompanied by a corresponding rise in hexokinase activity, may represent a compensatory response that enhances glucose-phosphorylation capacity during luteolysis. This alteration may help maintain the availability of glucose-6-phosphate for downstream glycolytic and pentose phosphate pathway reactions. Notably, downstream PPP metabolites remained unchanged, indicating that the metabolic alteration is concentrated at the level of the early oxidative PPP rather than the entire pathway. Direct isotope-tracing studies will be required to determine whether these changes reflect altered glucose utilisation or pathway flux.

Traditionally, G6PD, together with NADPH produced via the G6PD-mediated pentose phosphate pathway, is believed to primarily function as an antioxidant and exert pro-survival activity. However, we found that pharmacological or genetic inhibition of G6PD attenuated oxidative stress, ferroptosis markers, and luteal cell death during PGF2α-induced luteolysis. These findings align with previous studies showing protective effects of PPP inhibition in other models of oxidative injury [42–44], including the seminal observation by Dixon et al. that 6-AN mitigated erastin-induced ferroptosis [45]. These observations raise the possibility that under high oxidative stress, G6PD-derived NADPH may contribute to pro-oxidant effects rather than exclusively supporting antioxidant defence. Despite increased G6PD activity during luteal regression, NADPH levels remained low, whereas NADP⁺ and ROS levels were elevated, indicating an imbalance between NADPH generation and consumption. Previous studies in other cell types have suggested that G6PD-derived NADPH may fuel NOX2/NOX4-mediated ROS production [46]. However, because NOX activity was not directly measured in the present study, whether this mechanism operates in luteal cells remains unresolved.

PGF2α upregulated NOX2 and NOX4 in both luteal tissue and primary luteal cells. While NOX2 is a highly regulated, inducible oxidase, and NOX4 is constitutively active [28,47,48], both are significant sources of cellular ROS. To investigate their collective contribution and minimise potential functional compensation between the two isoforms [49,50], we adopted a simultaneous NOX2/NOX4 knockdown strategy. This is further supported by evidence that both isoforms can drive ferroptosis [30,51–54]. In luteal tissue, combined NOX2/NOX4 knockdown attenuates PGF2α-induced oxidative stress, G6PD hyperactivation, and ferroptosis-related alterations, ultimately delaying structural luteal regression. These *in vivo* findings were recapitulated in primary luteal cells, where simultaneous silencing of NOX2 and NOX4 similarly attenuated oxidative stress and G6PD activation. Treatment with the ROS scavenger NAC or the ferroptosis inhibitor Fer-1 also suppressed PGF2α-induced G6PD activation and, interestingly, reduced NOX2/NOX4 expression. These data suggest that G6PD hyperactivation is an oxidative-stress-responsive metabolic alteration associated with NOX2/NOX4-related redox signalling.

Beyond G6PD-specific effects, the restoration of PKM enzymatic activity following NOX2/NOX4 knockdown highlights a broader interplay between oxidative stress and glycolytic regulation, consistent with the known redox sensitivity of PKM2 [55–57]. The lack of a corresponding increase in steady-state pyruvate or lactate levels, despite restored PKM activity, suggests a rapid channelling of pyruvate into downstream pathways like the tricarboxylic acid cycle, a possibility that requires metabolic flux analysis for confirmation.

Several limitations of this study should be acknowledged. First, the present study employed a simultaneous knockdown strategy for NOX2 and NOX4 to verify the overall role of NOX-mediated oxidative stress in luteolysis. This experimental design cannot dissect the specific individual contribution of each isoform, nor can it distinguish whether the observed protective effects are driven by NOX2 inhibition alone, NOX4 inhibition alone, or their combined suppression. This represents a major limitation of the current work. Elucidating the distinct functions and upstream/downstream regulatory mechanisms of NOX2 and NOX4 in luteal ferroptosis will require single-gene knockdown or knockout models in future investigations. Second, although G6PD inhibition attenuated ROS and ferroptosis, we did not perform direct NOX activity assays or gain-of-function G6PD experiments. Thus, the direct channelling of G6PD-derived NADPH to NOX enzymes remains a hypothesis for future investigation. Third, GPx4 and ACSL4 were used as ferroptosis-associated biomarkers, but their regulatory relationship in luteal cells was not investigated. In addition, mitochondrial ultrastructural changes were not examined. Future studies should address both issues to strengthen the mechanistic and morphological evidence for ferroptosis during luteolysis. Fourth, our findings indicate that aberrant G6PD expression correlates with Fe^2^⁺ accumulation, both of which are implicated in oxidative stress and ferroptosis. However, whether Fe^2^⁺ accumulation is directly caused by G6PD dysfunction or secondary to redox imbalance remains unknown and the specific mechanism is needed to further research. Finally, the role of G6PD was validated pharmacologically *in vivo* and genetically *in vitro*, future cell-type-specific G6PD knockdown models will be essential to confirm its precise tissue-specific function in the corpus luteum.

In summary, this study reveals that luteal regression is characterised by a coordinated interplay among NOX2/NOX4-associated oxidative stress, ferroptosis, and G6PD-associated metabolic remodelling. Under sustained oxidative stress, the hyperactivation of G6PD functionally contributes to ferroptotic damage rather than restoring redox homoeostasis. These findings provide a novel oxidative-metabolic framework for understanding corpus luteum regression.

## Supplementary Material

Supplementary Material Supplementary Materials.docx

Supplementary Material Author Checklist Full.pdf

## Data Availability

Derived data supporting the findings of this study are available from the corresponding authors upon request. The targeted metabolomics data generated in this study have been deposited in the MetaboLights repository under accession number MTBLS15040.
