# Supplementary material for: NOX2/NOX4-associated oxidative stress contributes to G6PD-associated metabolic remodeling and ferroptosis during mice luteolysis
Source: Redox Rep. 2026 Sep 4;31(1):2724653. doi: 10.1080/13510002.2026.2724653 (PMC13552298; doi:10.1080/13510002.2026.2724653)
Supplement: Supplementary file 2 [file YRER_A_2724653_SM0752.docx]

**1. Materials and methods**

1.1 Immunohistochemistry

After deparaffinization and rehydration, antigen retrieval was performed in citrate buffer (pH 6.0) by microwave heating. Sections were blocked with 3% (v/v) H₂O₂ and 5% (w/v) BSA and then incubated overnight at 4 °C with primary antibodies against ACSL4 (Proteintech, Cat. 22401-1-AP; 1:500, v/v) and GPx4 (Abmart, Cat. T56959; 1:200, v/v), followed by incubation with an HRP-conjugated goat anti-rabbit IgG (H+L) secondary antibody (Proteintech, Cat. SA00001-2; 1:200, v/v) (37°C, 1 h). Signals were visualized using DAB (Zhongshan Golden Bridge, Cat. ZLI-9018) and counterstained with hematoxylin.

1.2 Pharmacological Inhibition

For *in vivo* experiments, mice received intraperitoneal injections of 6-aminonicotinamide (6-AN) (MCE, Cat. HY-W010342) at doses of 0, 5, 10, 30, and 50 mg/kg body weight at the time of 5 mg/kg PGF2α administration in the PGF2α-induced luteolysis model. Ovaries were harvested 24 h after PGF2α injection.

1.3 Western Blotting

The membranes were incubated overnight at 4 °C with primary antibodies against ACSL4 (Proteintech, Cat. 22401-1-AP), G6PD (Abcam, Cat. ab210702), and GPx4 (Abmart, Cat. T56959), each at a dilution of 1:1,000 (v/v). After washing, the membranes were incubated at room temperature for 1.5 h with an HRP-conjugated goat anti-rabbit IgG (H+L) secondary antibody (Proteintech, Cat. SA00001-2; 1:10,000, v/v)

1.4 Measurement of pyruvate and L-lactate levels

Tissue samples were homogenized and centrifuged. An aliquot of the supernatant was used for protein quantification. Another aliquot of the supernatant was mixed with assay reagents and transferred to 96-well plates. Absorbance readings were collected at 340 nm for pyruvate (Cat. G0807W), 530 nm for L-lactate (Cat. E-BC-K044-M). Pyruvate analysis kit was obtained from Suzhou Grace Biotechnology Co., Ltd. (Suzhou, China) and the L-lactate analysis kit was obtained from Elabscience Biotechnology Co., Ltd. (Wuhan, China).

1.5 Immunofluorescence staining

Cells grown on coverslips were fixed with paraformaldehyde, permeabilized with 0.5% (v/v) Triton X-100, and blocked with 5% (w/v) BSA. Cells were incubated overnight at 4 °C with primary antibodies against 3β-HSD (Cat. 15516-1-AP, Proteintech; 1:200, v/v) and StAR (Proteintech, Cat. 12225-1-AP; 1:200, v/v), followed by incubation with the corresponding fluorescent secondary antibody (Abcam, Cat. ab150080; 1:200, v/v) in the dark. The coverslips were mounted using an antifade mounting medium containing DAPI (Solarbio, Cat. S2110), and fluorescence images were acquired.

1.6 Ferrous ion fluorescence staining

Frozen ovarian tissue sections were incubated with 1 μM FerrOrange probe at 37 °C for 30 min and imaged using a confocal laser-scanning microscope. For cellular staining, cells were seeded in 24-well plates and treated with the indicated agents after attachment. After treatment, the cells were washed three times with PBS and incubated with 1 μM FerrOrange probe at 37°C for 30 min in the dark. Fluorescence images were then acquired using an inverted fluorescence microscope. FerrOrange was purchased from Dojindo Laboratories (Cat. F374).

1.7 Lipid peroxidation measurement

Cells were seeded in six-well plates and treated with the indicated agents after attachment. Cells were then incubated with 5 μM C11-BODIPY 581/591 in fresh serum-free medium at 37 °C for 30 min in the dark. After trypsinization, the cells were washed three times with serum-free medium to remove unbound probe. Fluorescence was measured using a BD flow cytometer with the FITC channel, and the mean fluorescence intensity (MFI) was analyzed using FlowJo software. C11-BODIPY was purchased from Dojindo Laboratories (Cat. L267).

1.8 Cell viability assay

Cell viability was measured by Cell Counting Kit-8 (CCK-8, Abbkine, Cat. BMU106).

**2. Result**

Supplemental figure 1

(A-B) Immunohistochemical staining of ACSL4 and GPX4 in ovarian tissues collected on day 9 of pregnancy (P9) and postpartum day 1 (PP1) (A), and in the control and PGF2α-treated groups (B). Insets show higher-magnification views of the boxed regions. Red arrows indicate positive staining. (C-D) Representative FerrOrange fluorescence staining of ferrous ions (Fe²⁺) in ovarian tissues collected on GD9 and PP1 (C), and in the control and PGF2α-treated groups (D). (n = 3 independent biological experiments)

Supplemental figure 2

(A) Schematic diagram of the experimental timeline. Luteogenesis was induced in vivo in 21-day-old female mice, followed by luteal cell isolation and subsequent PGF2α treatment in vitro. (B-C) Representative immunofluorescence images confirming the identity of the isolated cells by positive staining for the luteal cell markers STAR (red) and 3β-HSD (red). Nuclei are counterstained with DAPI (blue). Insets show higher-magnification views of the boxed regions. (D) Representative FerrOrange fluorescence staining of Fe²⁺ in luteal cell from the control, PGF2α-treated for 12 h, and PGF2α-treated for 24 h groups. (n = 3 independent biological experiments).

Supplemental figure 3

(A) PKM enzymatic activity in luteal tissues from the PGF2α-induced luteolysis model: AD-shNC versus AD-shNOX2/shNOX4 group. (B) L-Lactate content in luteal tissues from the PGF2α-induced luteolysis model: AD-shNC versus AD-shNOX2/shNOX4 group. (C) Pyruvic content in luteal tissues from the PGF2α-induced luteolysis model: AD-shNC versus AD-shNOX2/shNOX4 group. Data are shown as mean ± SD. Quantitative analyses were performed with n = 3 independent biological experiments for panels A-C. Unpaired two-tailed Student’s t-test were used for A-C. ns, not significant (*p* > 0.05); **p* < 0.05.

Supplemental figure 4

(A) Representative FerrOrange fluorescence images of intracellular Fe²⁺. (B) Representative flow-cytometric profiles and relative mean fluorescence intensity (MFI) quantification of oxidized C11-BODIPY for lipid peroxidation assessment. Cells were treated with 1μM PGF2α for 4 hours. Following co-treatment with shNC or shNOX2/NOX4 adenovirus for 48 hours before FerrOrange fluorescence. Cells were transduced with Ad-shNC or Ad-shNOX2/4 for 24 h, followed by treatment with 1 μM PGF2α for 12 h before C11-BODIPY analysis. Data are shown as mean ± SD. Statistical analyses for panels B were performed using one-way ANOVA followed by Tukey’s post hoc test. ns, not significant; **p* < 0.05, ***p* < 0.01, ****p* < 0.001. (n = 3 independent biological experiments).

Supplemental figure 5

(A) The schematic timeline outlines the key steps in establishing the PGF2α-induced luteolysis model, including treatment with 6-AN. (B) Illustration of the mechanism and molecular targets of 6-AN. (C) The enzymatic activities of G6PD were assayed following administration at doses of 0, 5, 10, 30, and 50 mg/kg. Data are shown as mean ± SD. Quantitative analyses were performed with n = 5 independent biological experiments for panel C. Panel C was analyzed using the one-way ANOVA followed by Tukey’s post hoc test. ns, not significant (p > 0.05); ***p* < 0.01; ****p* < 0.001.
